# ATG16L1 orchestrates interleukin-22 signaling in the intestinal epithelium via cGAS–STING

**DOI:** 10.1084/jem.20171029

**Published:** 2018-11-05

**Authors:** Konrad Aden, Florian Tran, Go Ito, Raheleh Sheibani-Tezerji, Simone Lipinski, Jan W. Kuiper, Markus Tschurtschenthaler, Svetlana Saveljeva, Joya Bhattacharyya, Robert Häsler, Kareen Bartsch, Anne Luzius, Marlene Jentzsch, Maren Falk-Paulsen, Stephanie T. Stengel, Lina Welz, Robin Schwarzer, Björn Rabe, Winfried Barchet, Stefan Krautwald, Gunther Hartmann, Manolis Pasparakis, Richard S. Blumberg, Stefan Schreiber, Arthur Kaser, Philip Rosenstiel

**Affiliations:** 1 Institute of Clinical Molecular Biology, Christian-Albrechts-University and University Hospital Schleswig-Holstein, Campus Kiel, Kiel, Germany; 2 Department of Internal Medicine I., Christian-Albrechts-University and University Hospital Schleswig-Holstein, Campus Kiel, Kiel, Germany; 3 Department of Gastroenterology and Hepatology, Tokyo Medical and Dental University, Tokyo, Japan; 4 Department of Medicine II, Klinikum Rechts der Isar, Technical University of Munich, Munich, Germany; 5 Division of Gastroenterology and Hepatology, Department of Medicine, Addenbrooke’s Hospital, University of Cambridge, Cambridge, England, UK; 6 Institute of Biochemistry, Kiel University, Kiel, Germany; 7 Institute for Genetics, CECAD, University of Cologne, Cologne, Germany; 8 Institute of Clinical Chemistry and Clinical Pharmacology, University Hospital Bonn, Bonn, Germany; 9 Department of Nephrology and Hypertension, University Hospital Schleswig-Holstein, Campus Kiel, Kiel, Germany; 10 Gastroenterology Division, Department of Medicine, Brigham and Women’s Hospital, Harvard Medical School, Boston, MA

## Abstract

A coding variant of the inflammatory bowel disease (IBD) risk gene *ATG16L1* has been associated with defective autophagy and deregulation of endoplasmic reticulum (ER) function. IL-22 is a barrier protective cytokine by inducing regeneration and antimicrobial responses in the intestinal mucosa. We show that ATG16L1 critically orchestrates IL-22 signaling in the intestinal epithelium. IL-22 stimulation physiologically leads to transient ER stress and subsequent activation of STING-dependent type I interferon (IFN-I) signaling, which is augmented in *Atg16l1*^ΔIEC^ intestinal organoids. IFN-I signals amplify epithelial TNF production downstream of IL-22 and contribute to necroptotic cell death. In vivo*,* IL-22 treatment in *Atg16l1*^ΔIEC^ and *Atg16l1*^ΔIEC^/*Xbp1*^ΔIEC^ mice potentiates endogenous ileal inflammation and causes widespread necroptotic epithelial cell death. Therapeutic blockade of IFN-I signaling ameliorates IL-22–induced ileal inflammation in *Atg16l1*^ΔIEC^ mice. Our data demonstrate an unexpected role of *ATG16L1* in coordinating the outcome of IL-22 signaling in the intestinal epithelium.

## Abstract (precis)

Dysregulated autophagy and ER stress are involved in the etiology of human IBD. Aden et al. show that loss of ATG16L1 function renders intestinal epithelial cells vulnerable to IL-22–induced ER stress and necroptosis via STING signaling, which induces ileal inflammation in vivo.

## Graphical Abstract

**Figure:**
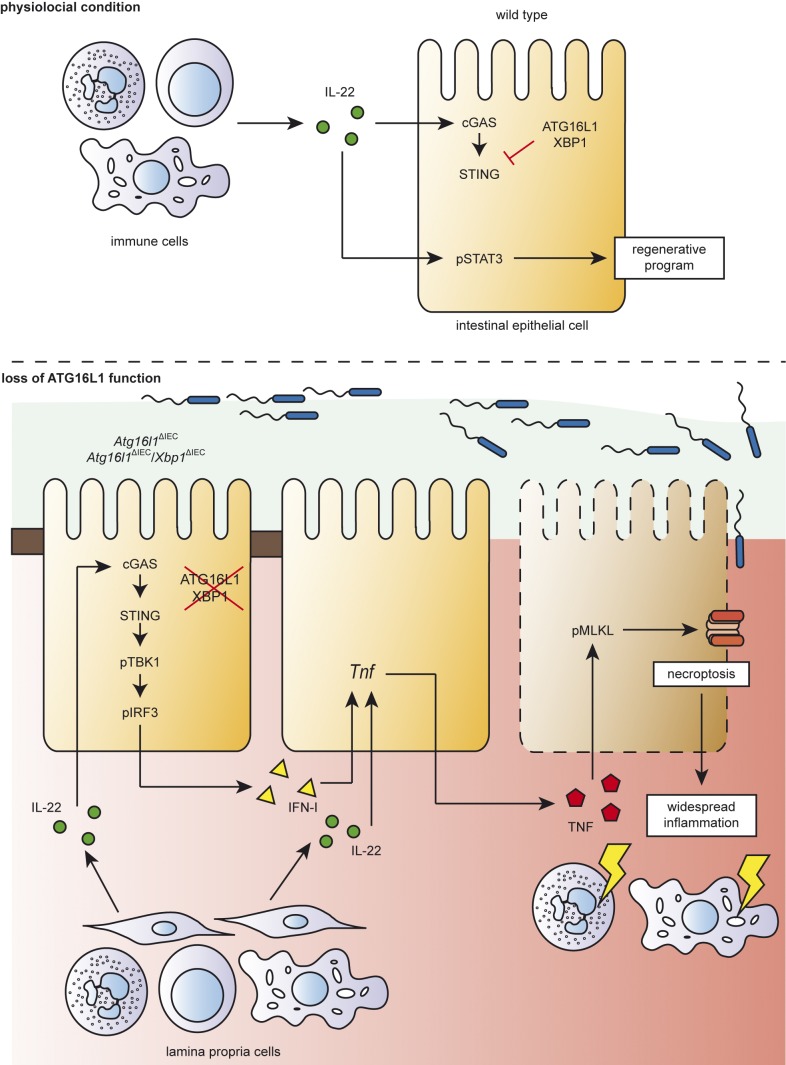

## Introduction

A defective intestinal barrier, impaired host microbial crosstalk, and a chronic preponderance of proinflammatory cytokines and T cell–mediated immunity are key elements of the formal etiology of human inflammatory bowel disease (IBD). Genome-wide association studies have identified a plethora of >200 risk loci predisposing to disease manifestation (Jostins et al., 2012) that cluster in distinct molecular pathways, including autophagy (Hampe et al., 2007), ER stress signaling, and innate immune sensing (Franke et al., 2010; Jostins et al., 2012). Although there is a strong genetic overlap observed between ulcerative colitis (UC) and Crohn’s disease (CD), variants in autophagy genes only affect CD patients and have been associated with Paneth cell defects (Cadwell et al., 2008).

Autophagy is a process allowing the orderly degradation and recycling of cellular components. Insufficient ATG16L1-mediated autophagy, e.g., by harboring the CD T300A risk allele, renders epithelial cells more susceptible to bacteria and virus-induced inflammation (Cadwell et al., 2010; Lassen et al., 2014). Autophagy is also closely intertwined to the unfolded protein response (UPR), elicited from the endoplasmic reticulum (Adolph et al., 2013; Deuring et al., 2014; Tschurtschenthaler et al., 2017). The importance of this crosstalk has been emphasized by the finding that mice, which are double deficient for the UPR transcription factor *Xbp1* and *Atg16l1* in the intestinal epithelium, develop a spontaneous transmural and fistulizing ileal inflammation reminiscent of human CD (Adolph et al., 2013).

IL-22 belongs to the family of IL-10 cytokines, is secreted from immune cells, including innate lymphoid cells, T cells, and neutrophilic granulocytes, and directly targets intestinal epithelial cells (Sonnenberg et al., 2011; Mielke et al., 2013; Zindl et al., 2013; Aden et al., 2016). IL-22 contributes to intestinal immune response toward pathogen infection (Zheng et al., 2008; Hernández et al., 2015) and epithelial wound healing (Pickert et al., 2009), namely via instruction of epithelial proliferation and the induction of secreted antimicrobial proteins (Huber et al., 2012; Pham et al., 2014; Lindemans et al., 2015). Hence, IL-10 itself has been described to diminish epithelial ER stress, which involves the induction of chaperones (Hasnain et al., 2013, 2014). Thus, we hypothesized that IL-22 could beneficially modulate cellular function and epithelial homeostasis in situations of defective autophagy or ER stress.

In this study, we report that the interplay of the UPR and autophagy pathways orchestrate a physiological dichotomy of IL-22 signaling in the intestinal epithelium. We demonstrate that epithelial IL-22 stimulation leads to release of cytosolic dsDNA and a consecutive self-activation of the cGAS–STING–IFN-I pathway and necroptosis, which is aggravated by autophagy and ER stress deficiency. Mechanistically, this process involves induction of epithelial TNF and mixed lineage kinase domain-like protein (MLKL), a core protein of the necroptosis machinery. We show that IL-22 treatment in animals carrying a conditional deletion of *Atg16l1* in the intestinal epithelium leads to induction of inflammation upon dextran sodium sulfate (DSS) irritant challenge, rather than protection. Collectively, our data identify unexpected roles of (1) IL-22 in engaging the cGAS–STING pathway to promote a proinflammatory, necroptotic response in intestinal epithelial cells and of (2) the key autophagy molecule *ATG16L1* in balancing the fate of such IL-22 signals in the intestine.

## Results

### The interplay of ATG16L1-mediated autophagy and ER stress resolution governs the cellular fate of IL-22 signaling

To investigate the role of ATG16L1-mediated autophagy on IL-22 signaling, small intestinal organoids of villin (V)-cre^+^; *Atg16l1*^fl/fl^ (hereafter called *Atg16l1*^ΔIEC^) or villin (V)-cre^−^ *Atg16l1*^fl/fl^ (hereafter called *Atg16l1*^fl/fl^) were cultured in the presence of rmIL-22 (1, 10, and 100 ng/ml).

**Figure 1.**
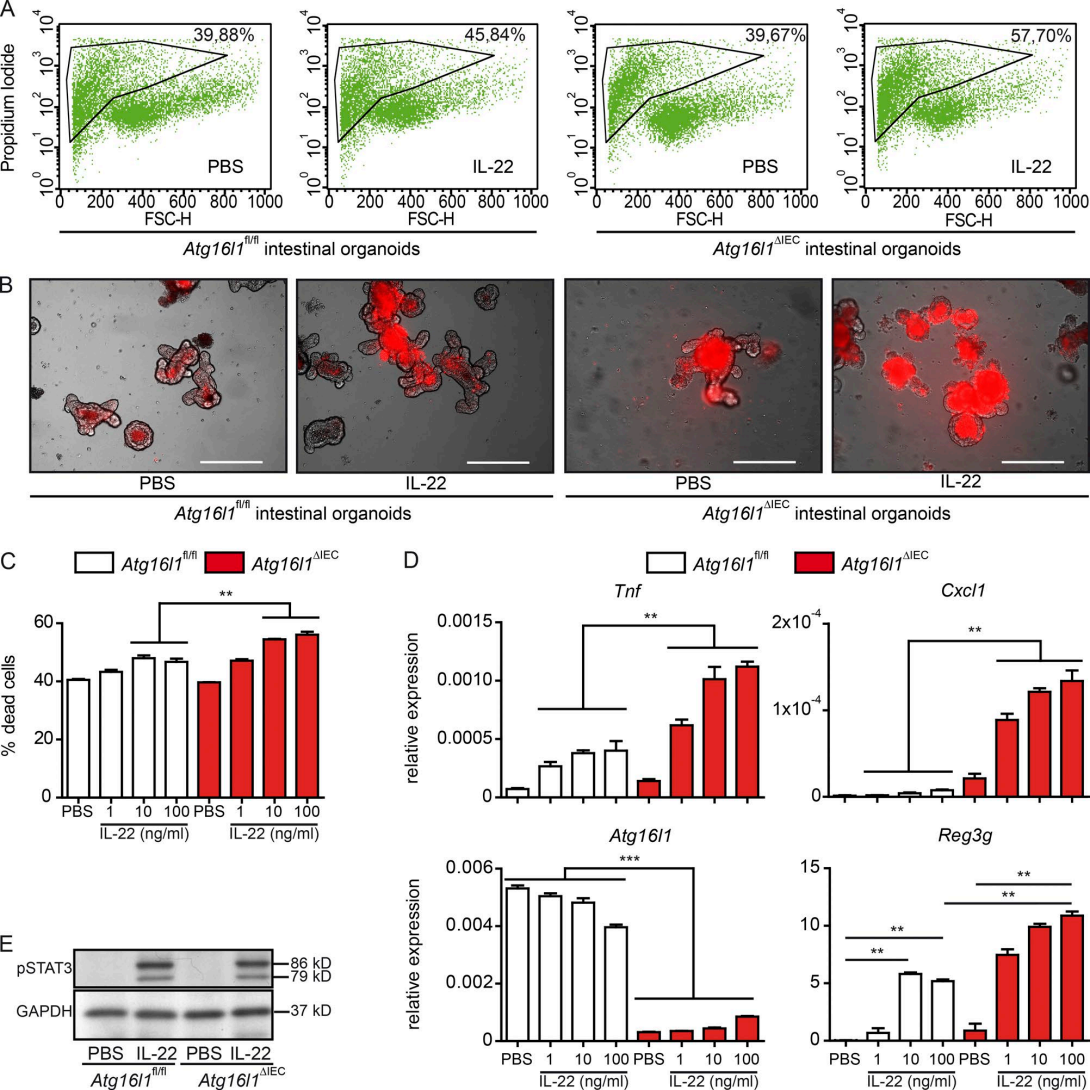
**IL-22 induces cell death and a proinflammatory signature in Atg16l1-deficient intestinal organoids. (A)** Representative FACS plots of PI-stained dissociated cells from intestinal organoids (*Atg16l1*^fl/fl^, *Atg16l1*^ΔIEC^), treated with rmIL-22 (100 ng/ml) for 24 h. **(B)** Representative pictures of intestinal organoids (*Atg16l1*^fl/fl^, *Atg16l1*^ΔIEC^), treated with rmIL-22 (100 ng/ml) for 24 h, stained with PI. Bars, 200 µm. **(C)** Flow cytometry assessment of dead cells from intestinal organoids (*Atg16l1*^fl/fl^, *Atg16l1*^ΔIEC^) stimulated with rmIL-22 (1, 10, or 100 ng/ml) for 24 h using PI (*n* = 3 each). **(D)** mRNA expression of *Tnf*, *Cxcl1*, *Atg16l1*, and *Reg3g* in small intestinal organoids (*Atg16l1*^fl/fl^, *Atg16l1*^ΔIEC^) treated with rmIL-22 (1, 10, or 100 ng/ml) for 24 h as assessed by qPCR (*n* = 4 each). **(E)** Western blot analysis from intestinal organoids (*Atg16l1*^fl/fl^, *Atg16l1*^ΔIEC^) treated with rmIL-22 (100 ng/ml) for 30 min, probed against pSTAT3 and GAPDH. Illustrated are representative data of three independent experiments. Significance determined using Mann-Whitney test and expressed as the mean ± SEM. **, P < 0.01; ***, P < 0.001.

IL-22 (100 ng/ml) treatment induced cell death in *Atg16l1*^ΔIEC^, but not in *Atg16l1*^fl/fl^ organoids (Fig. 1, A–C), and strongly induced *Tnf* and *Cxcl1* expression in *Atg16l1*^ΔIEC^ organoids, consistent with the absence of ATG16L1’s function inducing a proinflammatory tone (Fig. 1 D; Adolph et al., 2013). IL-22–induced STAT3 signaling appeared unaltered between genotypes although induction of the STAT3 downstream target *Reg3g* was increased in *Atg16l1*^ΔIEC^ cultures (Fig. 1 E). Chronic ER stress in intestinal organoids derived from the small intestine of villin (V)-cre^+^; *Xbp1*^fl/fl^ (hereafter called *Xbp1*^ΔIEC^) also rendered cells susceptible to IL-22–induced proinflammatory gene expression compared with villin (V)-cre^−^; *Xbp1*^fl/fl^ (hereafter called *Xbp1*^fl/fl^) organoids (Fig. S1 A; Kaser et al., 2008). Hence, we studied whether IL-22 is directly involved in epithelial ER stress induction under physiological conditions. IL-22 induced ER stress in vivo (Fig. S1 B) and in vitro and exacerbated ER stress induced by tunicamycin (TM) in HT-29 IECs (Fig. S1, C and D) and in *C57BL/6* (WT) small intestinal organoids (Fig. S1 E). Intestinal organoids from *Atg16l1*^ΔIEC^ (Fig. S1 F) or intestinal epithelial Caco-2 cells with a CRISPR/Cas9-guided deletion of *ATG16L1* (Fig. S1 G) exhibited an increased sensitivity to IL-22–induced ER stress as shown by increased *XBP1* splicing.

IL-22–dependent amplification of ER stress severely impaired epithelial wound healing and induced a proinflammatory phenotype in HT-29 IECs (Fig. S2, A–F). IL-22–mediated enhancement of chemically induced ER stress was dependent on STAT3 activation as pharmacological inhibition using the STAT3 inhibitor S3I-201 suppressed ER stress induction and reestablished epithelial wound healing. ER stress is known to be counterbalanced by autophagy (Adolph et al., 2013). Rapamycin, a known inducer of autophagy, prevented ER stress-dependent growth inhibition and proinflammatory cytokine production (Fig. S2, G and H) after IL-22 stimulation. Conversely, inhibition of autophagy by bafilomycin A1 (BafA) led to impaired wound healing and increased IL-8 secretion in cells treated with IL-22 (Fig. S2 J). To further delineate whether increased cell death in *Atg16l1*^ΔIEC^ intestinal organoids is specific to IL-22, we investigated the effect of IL-10. IL-10 induced epithelial cell death only in *Atg16l1*^ΔIEC^ organoids, but to a lesser extent compared with IL-22, whereas TM induced cell death in both genotypes, with significantly higher rates observed in *Atg16l1*^ΔIEC^ compared with *Atg16l1*^fl/fl^ organoids (Fig. S3, A and B), indicating a generally increased sensitivity to ER stress in ATG16L1-deficient intestinal epithelial cells. While we cannot fully exclude effects of ATG16L1 deficiency on other STAT3-dependent pathways (including IL-10), our data show that ATG16L1 and its interaction with ER stress responses strongly influences the outcome of IL-22–dependent signaling in intestinal epithelial cells.

### *ATG16L1* regulates IL-22–mediated transcriptional responses

**Figure 2.**
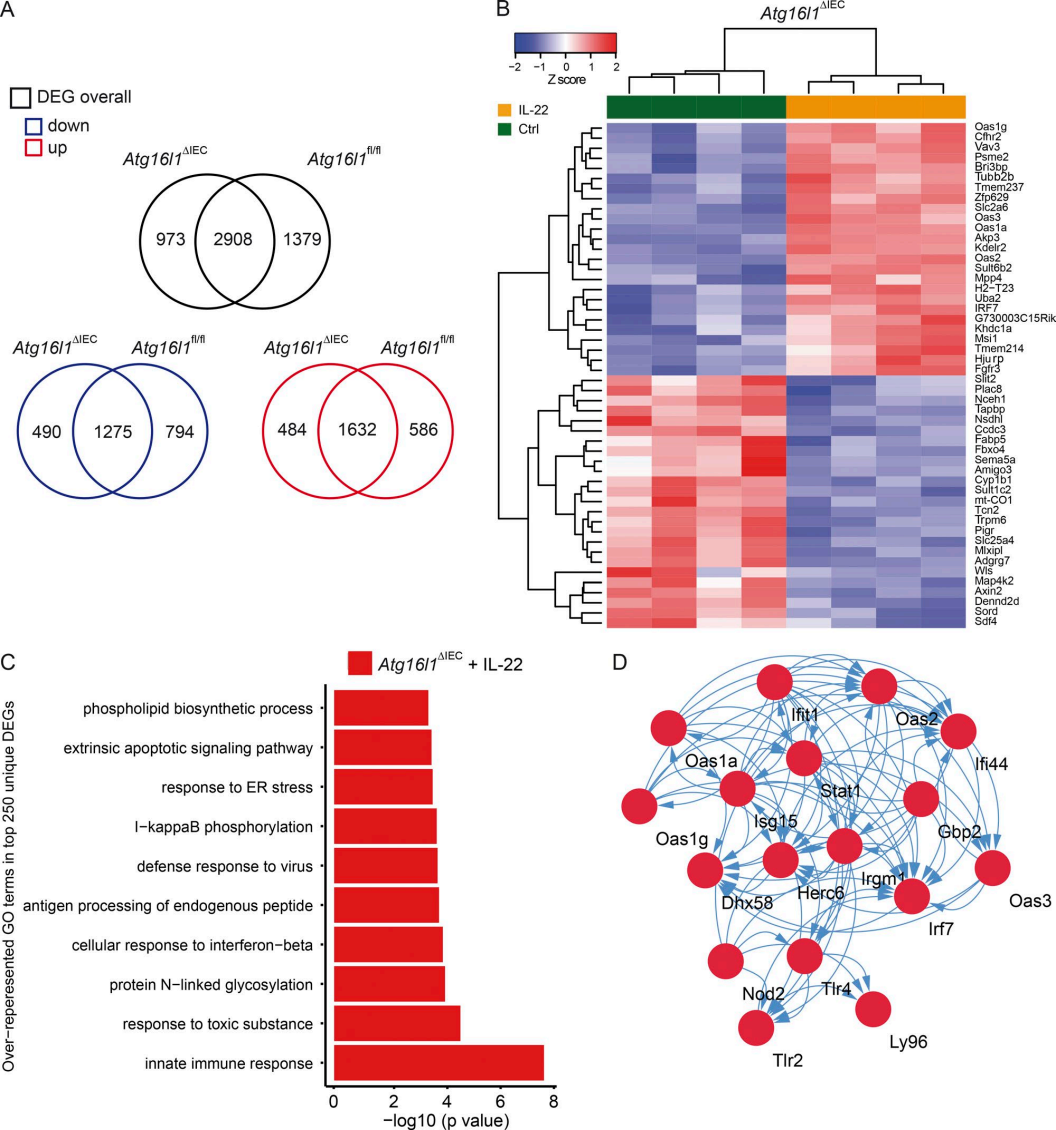
**Atg16l1 orchestrates an IL-22–dependent IFN-I signature in intestinal organoids. (A)** Venn diagram showing numbers of differentially expressed transcripts (overall, black) and significantly up-regulated (below, red) and down-regulated (below, blue) transcripts in small intestinal organoids (*Atg16l1*^fl/fl^, *Atg16l1*^ΔIEC^) in response to stimulation with IL-22 (10 ng/ml) for 24 h (*n* = 4 each). Differential expression was determined using RNA sequencing and the DESEQ2 algorithm. **(B)** Heat map showing clustering of top 25 up- and down-regulated genes in response to IL-22 (10 ng/ml) according to genotype. **(C)** Gene set enrichment (GO) analysis of top 250 uniquely up-regulated genes in IL-22–treated *Atg16l1*^ΔIEC^ intestinal organoids. **(D)** STRING-based network analysis of all genes contributing to the GO term “innate immune response” detected in C. Note a strong contribution of an IFN-I–related signature.

To analyze the transcriptomal program elicited by IL-22 in the absence or presence of ATG16l1, we performed RNA sequencing of small intestinal organoids derived from *Atg16l1*^ΔIEC^ or *Atg161*^fl/fl^ mice. In total, we found 2,908 differentially expressed genes and 586 (*Atg161*^fl/fl^) versus 484 (*Atg16l1*^ΔIEC^) uniquely up-regulated and 794 (*Atg16l1*^fl/fl^) versus 490 (*Atg16l1*^ΔIEC^) uniquely down-regulated genes in response to IL-22 (10 ng/ml for 24 h; Fig. 2 A and Tables S1–S6). The top 25 uniquely up- and down-regulated genes showed a surprising number of up-regulated transcripts known to play a role in innate immunity and anti-viral response and included the IFN-I–dependent genes *Oas1g*, *Oas3*, *Oas1a*, and *Irf7* (Fig. 2 B). We next performed formal gene set enrichment analysis using InnateDB (Breuer et al., 2013) to identify specific GO terms (gene ontology of biological processes) associated with genotype and treatment. In the top 500 regulated transcripts, we demonstrate that only in IL-22–treated *Atg16l1*^ΔIEC^, but not *Atg16l1*^fl/fl^ organoids, a significant enrichment of up-regulated processes related to innate immunity, herpes simplex infection, and cellular response to IFN-β is present in addition to expected pathways (e.g., ER stress and NF-κB activation; Fig. 2 C). Using the STRING (Szklarczyk et al., 2015) database, we performed an interaction analysis of all genes included in the GO term “innate immune response,” which revealed a densely connected network of known IFN-stimulated genes (ISG), e.g., *Stat1*, *Ifit1*, *Irf7, Oas1g*, and *Oas2* genes (Fig. 2 D). Notably, ISG and related processes were not up-regulated in IL-22–treated *Atg16l1*^fl/fl^ intestinal organoids (Fig. S3, C and E). In the absence of IL-22 stimulation, we only detected one ISG (*Ifitm3)* to be significantly up-regulated in *Atg16l1*^ΔIEC^ compared with *Atg16l1^fl/fl^* intestinal organoids (Fig. S3, D and F), indicating the absence of an IFN-I signature at baseline. Thus, we conclude that the interplay of deficient autophagy and IL-22 signaling is characterized by a unique IFN-I gene signature in intestinal epithelial cells.

### Loss of *Atg16l1* potentiates IL-22–induced IFN-I expression via STING-dependent recognition of cytosolic dsDNA

**Figure 3.**
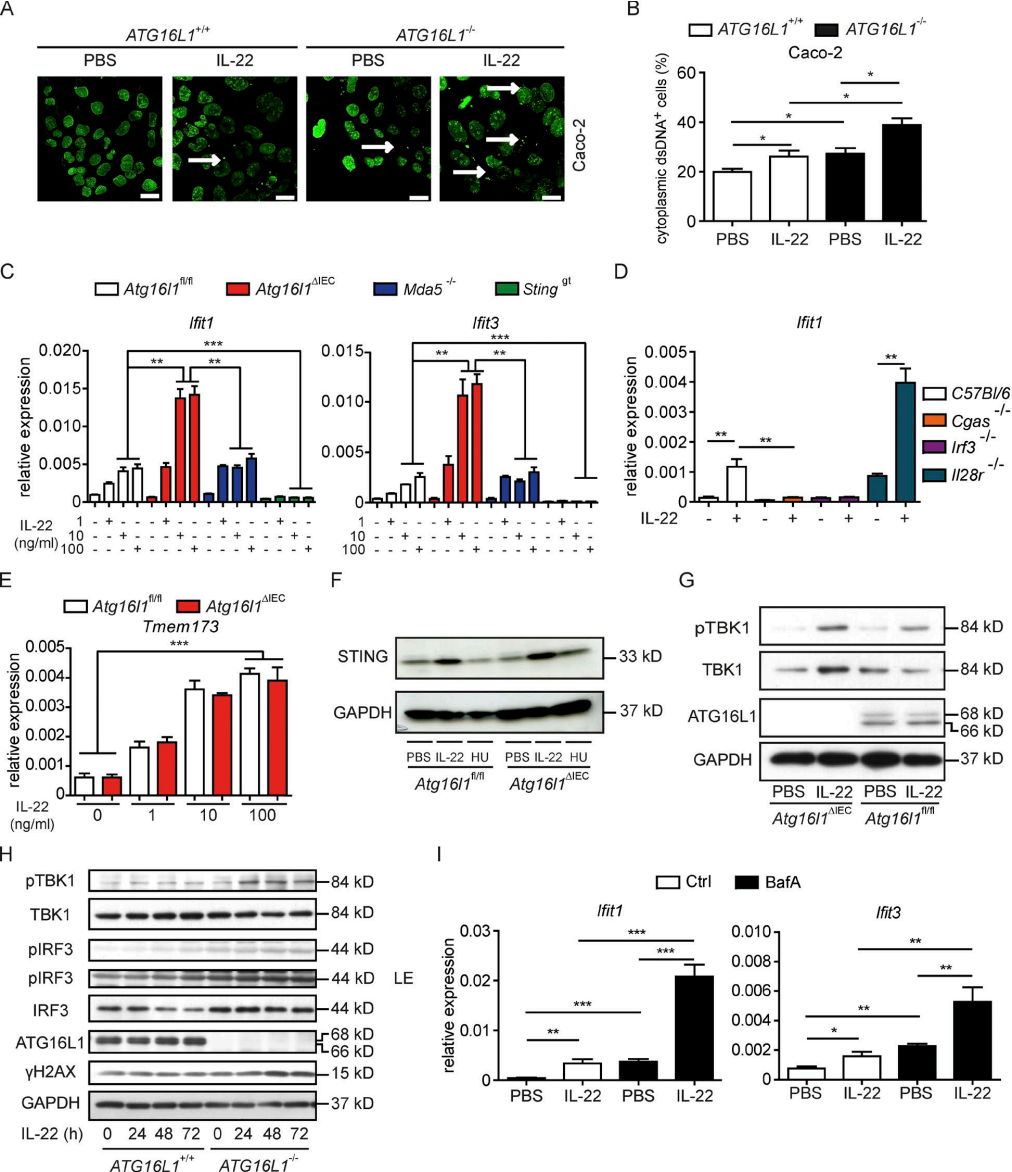
**ATG16L1 coordinates an IL-22–dependent IFN-I signature via STING signaling. (A and B)** Representative pictures (A) and quantification (B) of dsDNA in Caco-2 cells (*ATG16L1*^+/+^ vs. *ATG16L1*^−/−^) treated with rhIL-22 (100 ng/ml) for 24 h (*n* = 3 each). dsDNA was visualized in Caco-2 cells using an anti-dsDNA antibody (second antibody: Alexa Fluor 488–conjugated anti-mouse). Arrows indicate representative cytoplasmic dsDNA spots. Bars, 10 µm. **(C)** qPCR of *Ifit1* and *Ifit3* in intestinal organoids (*Atg16l1*^fl/fl^, *Atg16l1*^ΔIEC^, *Sting*^gt^, *Mda5*^−/−^) treated with or without rmIL-22 (1, 10, or 100 ng/ml) for 24 h (*n* = 3 each). **(D)** qPCR of *Ifit1* in intestinal organoids (*C57BL/6, Cgas^−/−^, Irf3^−/−^, Il28r^−/−^)* treated with rmIL-22 (100 ng/ml) or PBS for 24 h (*n* = 3 each). **(E)** qPCR of *Sting* in intestinal organoids (*Atg16l11*^fl/fl^, *Atg16l1*^ΔIEC^) treated with rmIL-22 (1, 10, or 100 ng/ml; *n* = 3 each). **(F)** Protein lysates from intestinal organoids (*Atg16l1*^fl/fl^, *Atg16l1*^ΔIEC^) treated with either rmIL-22 (100 ng/ml) or hydroxyurea (HU; 2 µM) for 24 h were subjected to immunoblot analysis against STING. **(G)** Western blot analyses from intestinal organoids (*Atg16l11*^fl/fl^, *Atg16l1*^ΔIEC^) treated with rmIL-22 (100 ng/ml) for 24 h. Lysates were probed against pTBK1, TBK1, ATG16L1, and GAPDH. **(H)** Protein lysates from Caco-2 cells (*ATG16L1*^+/+^ vs. *ATG16L1*^−/−^), treated with IL-22 (100 ng/ml) for indicated time points were subjected to immunoblot analysis against indicated proteins. LE: longer exposure. **(I)** qPCR of *Ifit1* and *Ifit3* in intestinal organoids (*C57BL/6J*) treated with rmIL-22 (100 ng/ml) and BafA (5 nM) for 24 h (*n* = 3 each). Results (A–I) represent at least two independent experiments. Significance determined using two-tailed Student’s *t* test and expressed as the mean ± SEM. *, P < 0.05; **, P < 0.01; ***, P < 0.001.

As cytosolic dsDNA is a strong inducer of ISG, and autophagy is required for removal of double-stranded (ds)DNA (Saitoh et al., 2009; Bartsch et al., 2017), we tested the hypothesis that IL-22 signaling may induce the release of dsDNA into the cytosol, which in turn may evoke the IFN signature. Using intestinal epithelial Caco-2 cells with a CRISPR/Cas9-guided deletion of *ATG16L1* (*ATG16L1^−/−^*), we observed a significant increase of nucleoids (i.e., cytosolic speckles staining positive for dsDNA; Bartsch et al., 2017) in response to IL-22 stimulation, which was augmented in *ATG16L1^−/−^* cells (Fig. 3, A and B). As initiation of IFN-I secretion upon cytosolic dsDNA is established by stimulator of IFN genes (STING), which is encoded by the *Tmem173* gene (Barber, 2015), we tested whether IL-22–dependent ISG induction is mediated via cyclic GMP-AMP synthase (cGAS, necessary for catalyzing cGAMP synthesis for STING activation)/STING. For this purpose, we derived intestinal organoids from *Sting*^gt^ mice, which harbor a single nucleotide variant (T596A) of *Tmem173*/STING that functions as a null allele and fails to produce detectable protein (Sauer et al., 2011). To exclude a role for cytosolic dsRNA as the origin of ISG regulation, we derived intestinal organoids from *Mda5*^−/−^ (melanoma differentiation-associated protein 5) mice, which carry a deletion of a crucial dsRNA helicase enzyme necessary for recognition of dsRNA (Wu et al., 2013). We showed that IL-22–induced ISG levels are indeed evoked by sensing of dsDNA, not RNA, as IL-22–induced ISG expression was completely abrogated in *Sting*^gt^, but not *Mda5*^−/−^ organoids (Fig. 3 C). The dependency of IL-22–initiated ISG induction on the cGAS–STING pathway was further confirmed using *Cgas*^−/−^ and *Irf3*^−/−^ (IFN regulatory factor 3, transcription factor downstream of STING) intestinal organoids, both of which showed absent ISG induction in response to IL-22 (Fig. 3 D). We excluded an amplifying role of type III IFNs for the induction of ISGs, as *Il28r*^−/−^ organoids showed an increased ISG expression in response to IL-22 (Fig. 3 D). IL-22 induced a comparable up-regulation of *Sting* on transcript (Fig. 3 E), as well as protein level (Fig. 3 F), both in *Atg16l1*^ΔIEC^ and *Atg16l1*^fl/fl^ organoids, indicating that the enhanced ISG induction cannot be explained by different *Sting* levels in the absence of ATG16L1. To further investigate whether *Atg16l1* deficiency increases downstream signaling of the cGAS–STING pathway, we analyzed the phosphorylation of TANK-binding kinase 1 (TBK1) in response to IL-22 stimulation, which was stronger in *Atg16l1*^ΔIEC^ organoids (Fig. 3 G) and *ATG16L1^−/−^* Caco-2 cells compared with their WT counterparts (Fig. 3 H).

Excessive STING activation in *Atg16l1*^ΔIEC^ organoids was dependent on defective autophagy as pharmacological inhibition of autophagy with BafA phenocopied the enhanced IL-22–induced ISG induction in WT organoids (Fig. 3 I). We further examined a potential interplay of ER stress and IL-22 signaling in amplifying epithelial DNA damage and subsequent ISG induction. Epithelial cells cotreated with IL-22 and TM exhibited increased epithelial DNA damage compared with cells treated only with IL-22 as shown by increased γH2AX staining and increased induction of *Cxcl10*. The latter effect could be rescued by STAT3 inhibition (Fig. S2, K and L).

### Systemic IL-22 treatment induces intestinal inflammation in *Atg16l1*^ΔIEC^ mice

**Figure 4.**
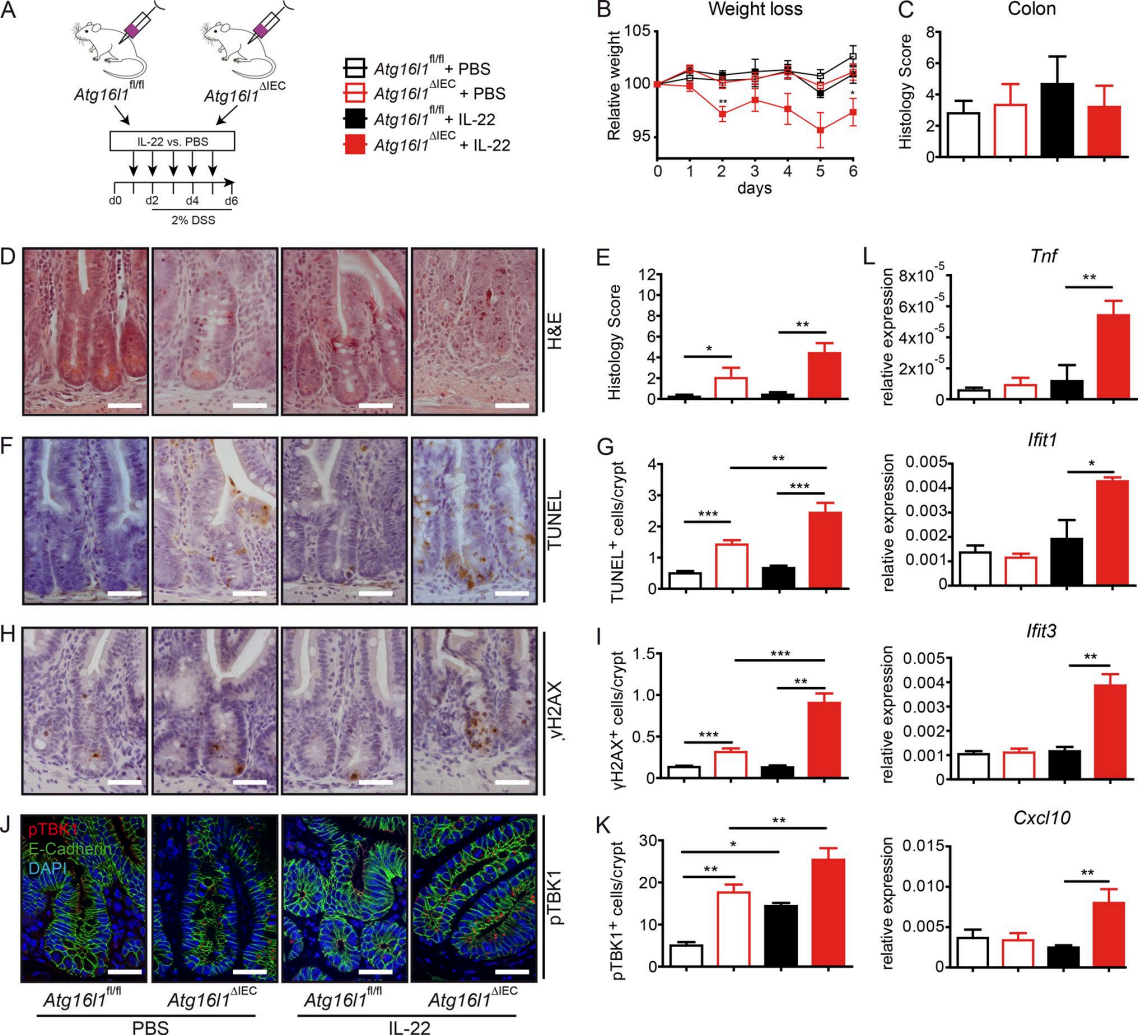
**IL-22 induces ileal inflammation in *Atg16l1*^ΔIEC^ mice. (A)** Stimulation scheme of *Atg16l1*^fl/fl^ and *Atg16l1*^ΔIEC^ mice (*n* = 5/5/5/5). Mice were treated with 2 µg/20 mg bodyweight of rmIL-22 i.p. every day over the course of 6 d. **(B)** Weight loss curve. **(C)** Statistical evaluation of the histological inflammation score in colon sections. **(D–K)** Histological evaluation of small intestinal sections with representative pictures and absolute quantification for H&E (D and E), TUNEL (F and G), and γH2AX (H and I; *n* = 5 each). Representative IF staining and statistical evaluation of small intestinal sections stained against pTBK1 (second antibody: Alexa Fluor 546–conjugated anti-rabbit; red) and counterstained with DAPI and anti-E-cadherin (second antibody: Alexa Fluor 488–conjugated anti-mouse; green; J and K; *n* = 5 each). For quantification, a minimum of 100 crypts/intestine were assessed in each treatment group by two independent observers. Bars, 100 µm. **(L)** Gene expression of *Tnf*, *Ifit1*, *Ifit3*, and *Cxcl10* from small intestinal crypts (*n* = 4 each). Results represent one experiment. Significance determined using two-tailed Student’s *t* test and expressed as the mean ± SEM. *, P < 0.05; **, P < 0.01; ***, P < 0.001.

To investigate the hypothesis that ATG16L1 coordinates protective IL-22 signaling in vivo, *Atg16l1*^fl/fl^ and *Atg16l1*^ΔIEC^ mice were treated with daily i.p. injections of recombinant IL-22 (2 µg/20 g bodyweight) for 6 d (Fig. 4 A) during a short course (3 d) of DSS in drinking water at a low concentration (2%). *Atg16l1*^ΔIEC^ mice presented with increased weight loss and heightened inflammatory disease activity index upon IL-22 treatment (Fig. 4 B). Using this mild treatment, we did not observe any induction of colonic inflammation, regardless of genotype or treatment (Fig. 4 C). Histological analysis revealed that the short course of DSS surprisingly led to significantly increased inflammatory cell infiltrates into the mucosa of the terminal ileum in *Atg16l1*^ΔIEC^ mice (Fig. 4, D and E), which was markedly enhanced by IL-22 administration. This was associated with increased levels of epithelial cell death and DNA damage as depicted by increased numbers of terminal dUTP nick end labeling (TUNEL)^+^ (Fig. 4, F and G) and γH2AX^+^ epithelial cells (Fig. 4, H and I), when compared with littermate *Atg16l1*^fl/fl^ mice. Of note, *Atg16l1*^ΔIEC^ mice also at baseline had slightly higher numbers of TUNEL^+^ epithelial cells and γH2AX^+^ epithelial cells, which may point to a vulnerable state of the epithelial lining in the absence of additional stressors. In line with increased STING activation, IL-22–treated *Atg16l1*^ΔIEC^ mice displayed a strong increase of punctate pTBK1 staining in the small intestinal crypt region (Fig. 4, J and K; and Fig. S4 A) and showed epithelial up-regulation of ISG (*Ifit1*, *Ifit3*, and *Cxcl10*) and *Tnf* as demonstrated by quantitative PCR (qPCR; Fig. 4 l).

### Systemic IL-22 treatment induces intestinal inflammation in *Atg16l1*^ΔIEC^/*Xbp1*^ΔIEC^ mice

**Figure 5.**
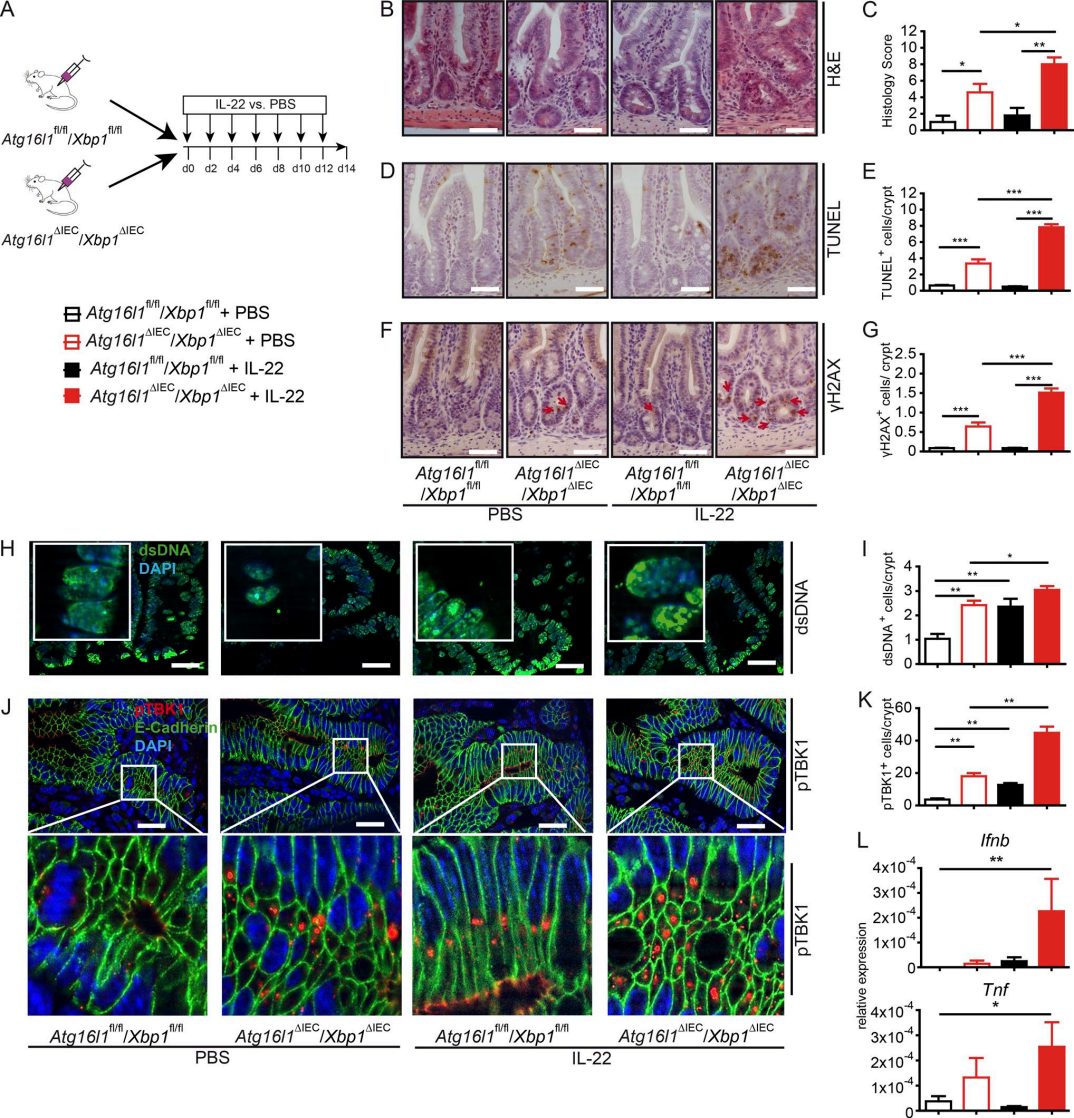
**IL-22 aggravates epithelial cell death–mediated inflammation in *Atg16l1*^ΔIEC^/*Xbp1*^ΔIEC^ mice. (A)** Treatment scheme of *Atg16l1*^fl/fl^/*Xbp1*^fl/fl^ and *Atg161*^ΔIEC^*/Xbp1*^ΔIEC^ mice (*n* = 7/7/7/6). **(B–K)** Histological evaluation of small intestinal sections with representative pictures and absolute quantification for H&E (B and C), TUNEL (D and E), and γH2AX (F and G; *n* = 5 each). Representative IF staining (including magnification inserts) and statistical evaluation of small intestinal sections stained against dsDNA (second antibody: Alexa Fluor 488–conjugated anti-mouse antibody; green; counterstained with DAPI; H and I) and pTBK1 (second antibody: Alexa Fluor 546–conjugated anti-rabbit; red), counterstained with DAPI and anti–E-cadherin (second antibody: Alexa Fluor 488–conjugated anti-mouse; green; J and K; *n* = 5 each). For quantification a minimum of 100 crypts/intestine were assessed in each treatment group. Bars, 100 µm. **(L)** qPCR of *Ifnb* and *Tnf* in ileal mucosa (*n* = 4 each). Results represent one experiment. Significance determined using two-tailed Student’s *t* test and expressed as the mean ± SEM. *, P < 0.05; **, P < 0.01; ***, P < 0.001.

We next investigated the impact of IL-22 on the inflammatory phenotype in mice carrying an epithelial deletion in *Atg16l1* and *Xbp1* (called *Atg16l1*^ΔIEC^/*Xbp1*^ΔIEC^ mice hereafter; Adolph et al., 2013). The line is known to develop spontaneous ileitis with signs of severe ER stress. *Atg16l1*^fl/fl^/*Xbp1*^fl/fl^ and *Atg16l1*^ΔIEC^/*Xbp1*^ΔIEC^ were treated systemically with recombinant IL-22 or PBS for 14 d (Fig. 5 A). As expected, *Atg16l1*^ΔIEC^/*Xbp1*^ΔIEC^ presented at baseline with significant ileal inflammation and cell death. This phenotype was aggravated upon IL-22 stimulation as demonstrated by histology (Fig. 5, B and C) and count of TUNEL^+^ (Fig. 5, D and E) and γH2AX^+^ (Fig. 5, F and G) cells. Intriguingly, IL-22 induced an increase in cytosolic dsDNA^+^ nucleoids (Fig. 5, H and I) and consecutive phosphorylation of TBK1^+^ (Fig. 5, J and K; and Fig. S4 B) cells along with increased small intestinal *Tnf* and *Ifnb* expression in IL-22–treated *Atg16l1*^ΔIEC^/*Xbp1*^ΔIEC^ mice (Fig. 5 L).

This corroborates the notion that ER stress and autophagy pathways control IL-22–dependent activation of STING-dependent signaling. Consistent with this, intestinal organoids double deficient for *Atg16l1* and *Ormdl3*, another ER stress regulatory gene, genetic variants of which confer genetic risk for IBD (McGovern et al., 2010), also exhibited significant up-regulation of ISG genes and *Tnf* induction in response to IL-22 stimulation (Fig. S4 C). Notably, epithelial ISG and *Tnf* induction appear confined to IL-22, as other cytokines of the IL-10 family (IL-10 and IL-19) were not able to fully phenocopy IL-22 effects in WT intestinal organoids (Fig. S4 D).

### cGAS–STING licenses TNF production and epithelial necroptosis in response to IL-22 stimulation

**Figure 6.**
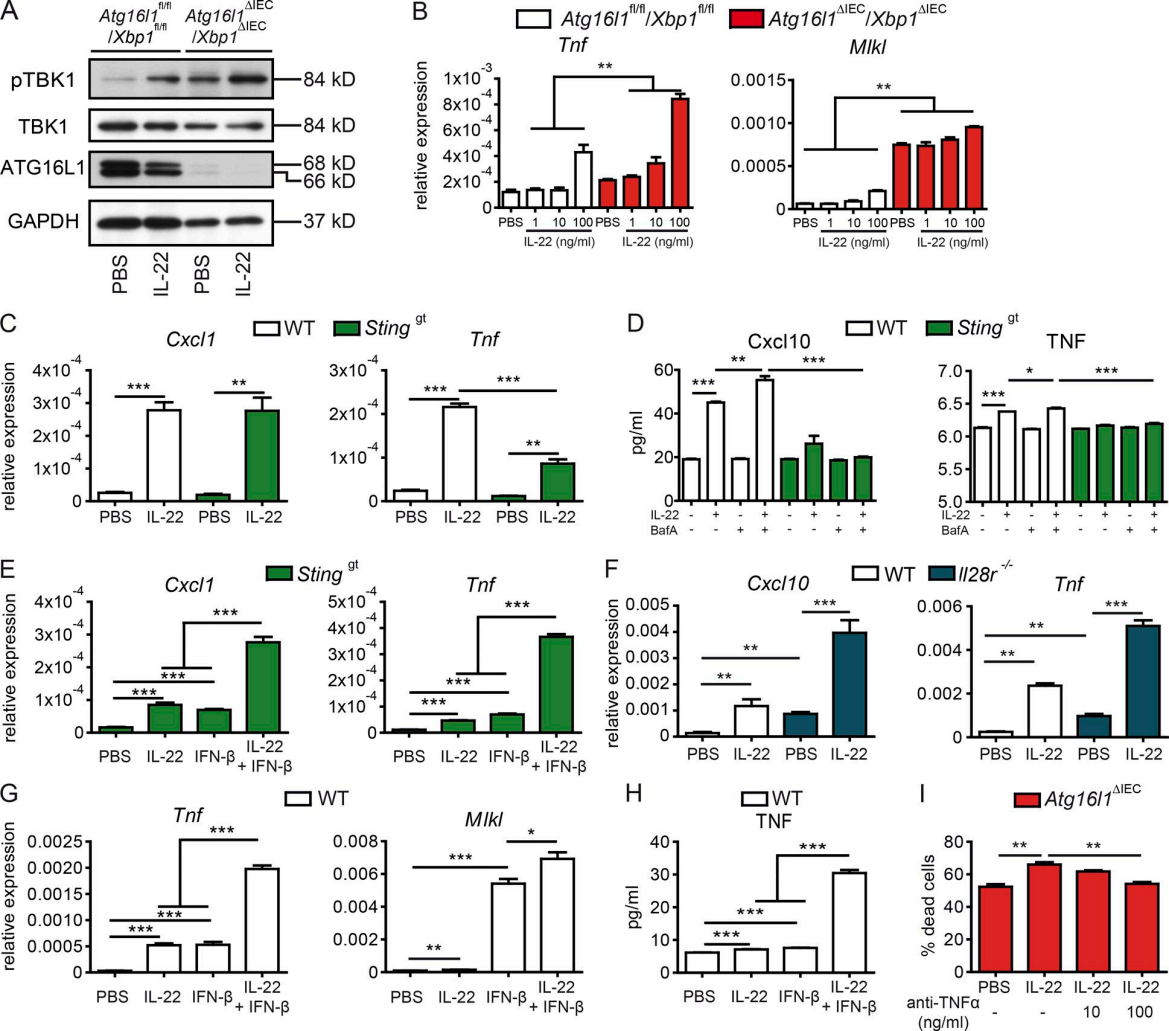
**STING and IFN-I signals synergize in TNF induction and necroptosis in intestinal epithelial organoids. (A)** Immunoblot analyses from protein lysates derived from *Atg16l1*^fl/fl^/*Xbp1*^fl/fl^ and *Atg16l1*^ΔIEC^/*Xbp1*^ΔIEC^ organoids stimulated with rmIL-22 (100 ng/ml) for 24 h and probed against pTBK1, TBK1, ATG16L1, and GAPDH. **(B)** Transcript levels of *Tnf* and *Mlkl* in small intestinal organoids (*Atg16l1*^fl/fl^/*Xbp1*^fl/fl^ and *Atg16l1*^ΔIEC^/*Xbp1*^ΔIEC^) treated with rmIL-22 (100 ng/ml) for 24 h as assessed by qPCR (*n* = 4 each). **(C)** Transcript levels of *Cxcl1*, *Tnf* in small intestinal organoids (*C57BL/6, Sting*^gt^) treated with rmIL-22 (100 ng/ml) for 24 h as assessed by qPCR (*n* = 4 each). **(D)** Concentration of CXCL10 and TNF in the supernatant of intestinal organoids (*C57BL/6, Sting*^gt^) treated with rmIL-22 (100 ng/ml), bafilomycin A (BafA; 5 nM) or both for 24 h, as detected via ELISA (*n* = 3 each). **(E)** qPCR of *Cxcl1*, *Tnf* in small intestinal organoids (*Sting*^gt^) treated with rmIL-22 (100 ng/ml) or IFN-β (1,000 IU/ml) or both for 24 h (*n* = 3 each). **(F)** qPCR of *Cxcl10*, *Tnf* in small intestinal organoids from *C57BL/6* or *Il28r^−/−^* mice treated with rmIL-22 (100 ng/ml) for 24 h (*n* = 3 each). **(G)** qPCR of *Mlkl* and *Tnf* in small intestinal organoids (*C57BL/6*) treated with rmIL-22 (100 ng/ml) or IFN-β (1,000 IU/ml) or both for 24 h (*n* = 3 each). **(H)** Concentration of TNF in the supernatant of intestinal organoids (*C57BL/6*) treated with rmIL-22 (100 ng/ml) or IFN-β (1,000 IU/ml) or both for 24 h, as detected via ELISA. **(I)** Assessment of dead cells from intestinal organoids (*Atg16l1*^ΔIEC^) stimulated with rmIL-22 (100 ng/ml) for 24 h in the absence or presence of anti-TNF antibody (10 and 100 ng/ml; *n* = 3 each). Results represent two independent experiments. Significance determined using two-tailed Student’s *t* test and expressed as the mean ± SEM. *, P < 0.05; **, P < 0.01; ***, P < 0.001.

Increased levels of TNF have been reported in the context of impaired ER stress and autophagy (Adolph et al., 2013), and TNF-induced cell death in *Atg16l1*^ΔIEC^ intestinal organoids depends on MLKL, which is a central regulator of necroptosis (Matsuzawa-Ishimoto et al., 2017). We therefore investigated whether (1) STING signaling is involved in epithelial TNF production downstream of IL-22 and (2) whether STING-amplified TNF production contributes to cell death in *Atg16l1*^ΔIEC^ or *Atg16l1*^ΔIEC^/*Xbp1*^ΔIEC^ intestinal organoids. We found that TBK1 phosphorylation upon IL-22 stimulation was increased in *Atg16l1*^ΔIEC^/*Xbp1*^ΔIEC^ intestinal organoids suggesting increased STING activation (Fig. 6 A). IL-22 led to a concentration-dependent up-regulation of *Tnf* and *Mlkl* expression (Fig. 6 B). We therefore hypothesized that cGAS–STING activation in response to IL-22 stimulation may orchestrate downstream expression of TNF. Supporting this hypothesis, IL-22–induced TNF expression (Fig. 6, C and D) was significantly reduced in *Sting^gt^* organoids, whereas vice versa cotreatment of *Sting^gt^* with IL-22 and IFN-β significantly increased *Tnf* and *Cxcl10* expression (Fig. 6 E). To delineate whether type III IFNs (e.g., IFN-λ) are involved in the IL-22 amplification loop to induce TNF, we stimulated intestinal organoids from WT or mice deficient for *Il28r*, the receptor for IFN-λ, with IL-22. IL-22–induced expression of *Cxcl10* and *Tnf* were increased in *Il28r*^−/−^ organoids, indicating that IFN-λ is not involved in amplifying *Tnf* induction (Fig. 6 F), but rather dampens inflammatory epithelial responses, as described previously (Chiriac et al., 2017). The enhancing effect of IL-22 and IFN-I on TNF induction was further confirmed in WT organoids as costimulation with IL-22, and IFN-β showed significantly increased *Tnf* and *Mlkl* gene expression (Fig. 6 G) and increased TNF protein concentration (Fig. 6 H).

To mechanistically prove that TNF is a downstream mediator of IL-22–induced cell death, we stimulated *Atg16l1*^ΔIEC^ intestinal organoids with IL-22 in the presence or absence of murine anti-TNF neutralizing antibody. We observed a dose-dependent protective effect of anti-TNF treatment, indicating that TNF acts as a downstream factor of IL-22–induced cell death (Fig. 6 I).

**Figure 7.**
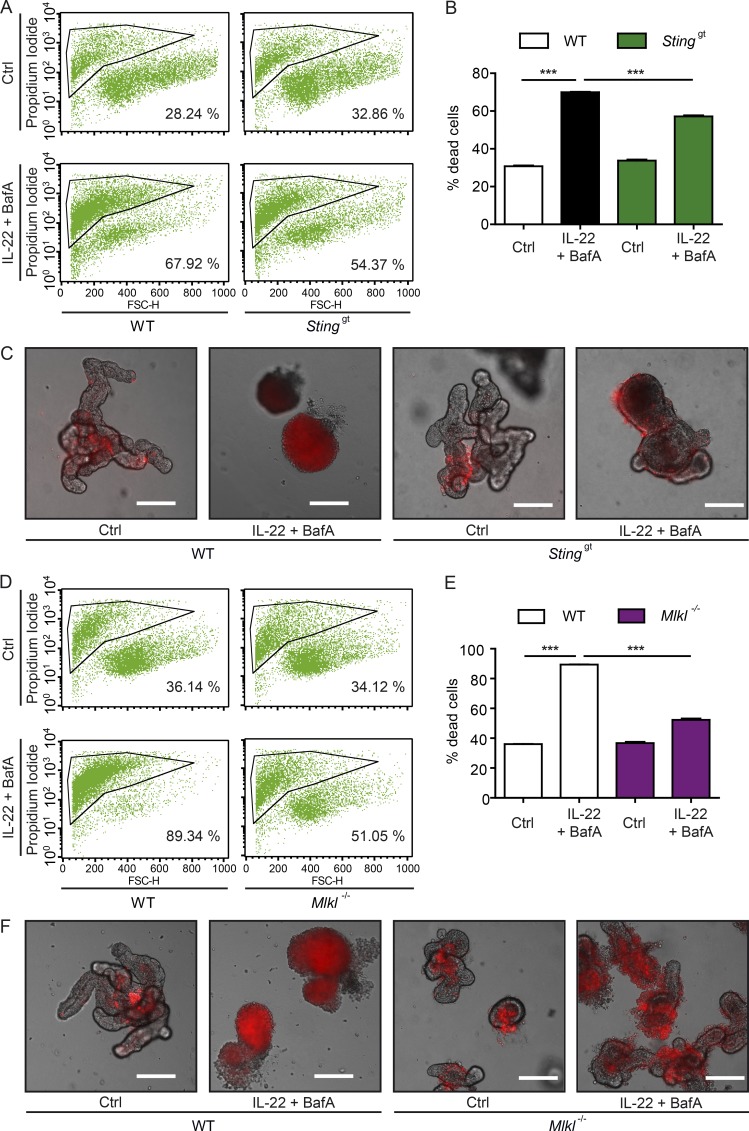
**IL-22–induced epithelial cell death depends on STING and MLKL. (A and B)** Representative FACS plots (A) and flow cytometry analysis (B) of cell death of intestinal organoids (WT, *Sting*^gt^), treated with rmIL-22 (100 ng/ml) and BafA (5 nM) for 24 h and staind with PI (*n* = 3 each). **(C)** Representative pictures of intestinal organoids (WT, *Sting*^gt^), treated with rmIL-22 (100 ng/ml) and BafA (5 nM) for 24 h. Bars, 200 µm. **(D and E)** Representative FACS plots (D) and flow cytometry analysis (E) of cell death of intestinal organoids (WT, *Mlkl*^−/−^), treated with rmIL-22 (100 ng/ml) and BafA (5 nM) for 24 h and stained with PI (*n* = 3 each). **(F)** Representative pictures of intestinal organoids (WT, *Mlkl*^−/−^), treated with rmIL-22 (100 ng/ml) and BafA (5 nM) for 24 h. Bars, 200 µm. Results represent two independent experiments. Significance determined using two-tailed Student’s *t* test and expressed as the mean ± SEM. *, P < 0.05; **, P < 0.01; ***, P < 0.001.

We next elucidated whether STING signaling is involved in IL-22–induced cell death in the context of impaired autophagy. We thus stimulated WT or *Sting*^gt^ intestinal organoids with IL-22 and BafA and assessed cell death in intestinal organoids by flow cytometry. *Sting^gt^* organoids were significantly protected from IL-22^+^BafA–induced cell death compared with WT organoids (Fig. 7, A–C). Since IFN-I induces MLKL expression (Günther et al., 2016), a central regulator of necroptosis, we investigated whether IL-22/STING–induced cell death depends on MLKL. IL-22^+^BafA–induced cell death was significantly lower in *Mlkl^−/−^* compared with WT intestinal organoids (Fig. 7, D–F). Thus, consistent with a recent report (Legarda et al., 2016) we conclude that IL-22/STING–dependent cell death involves MLKL-dependent necroptosis.

### IFN-I synergistically contributes to the IL-22–induced ileitis of *Atg16l1*^ΔIEC^ mice

**Figure 8.**
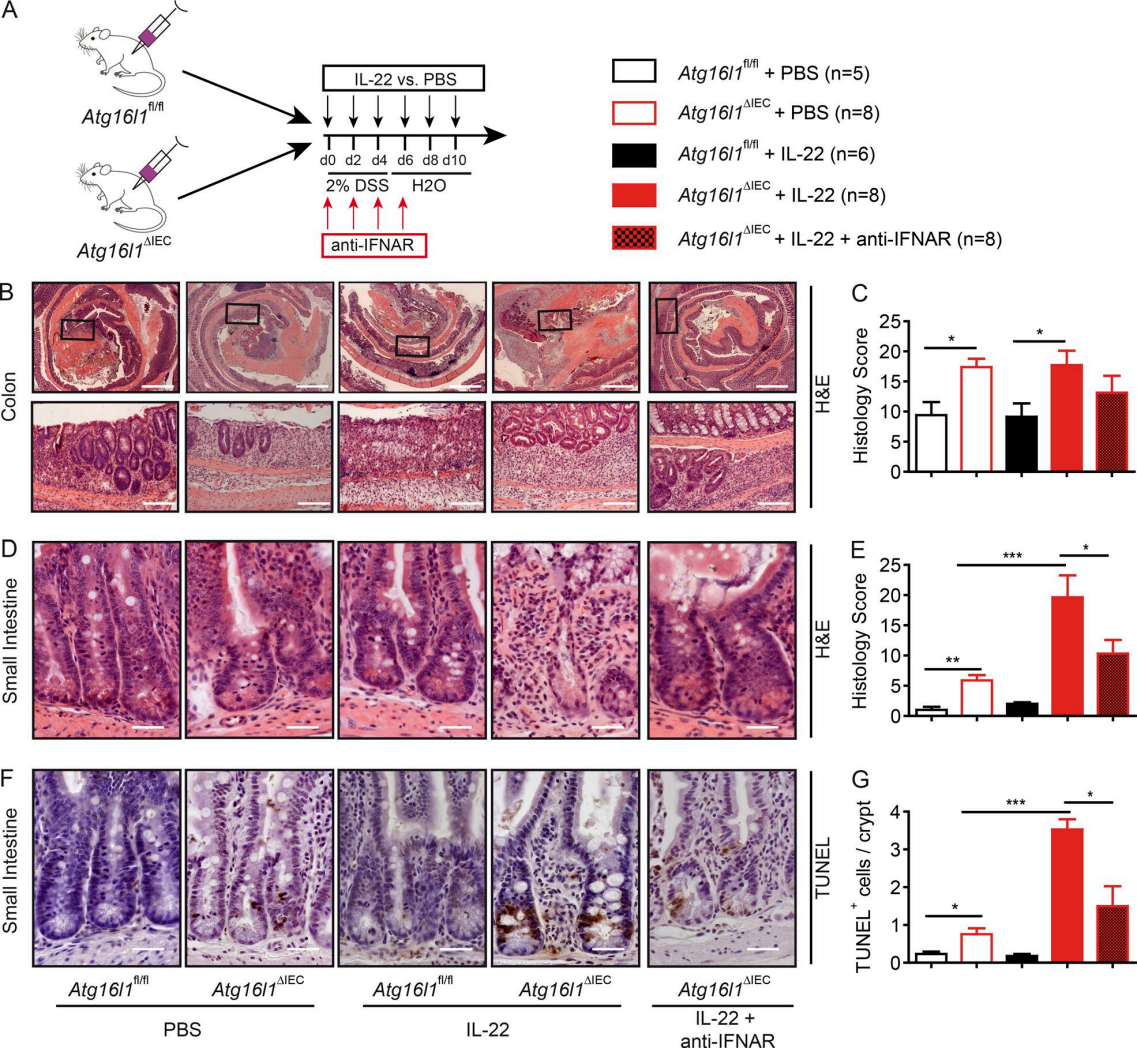
**IFN-I signals contribute to IL-22–induced ileitis in *Atg16l1^ΔIEC^* mice. (A)** Stimulation scheme of *Atg16l1*^fl/fl^ and *Atg16l1*^ΔIEC^ mice treated with rmIL-22 and anti-IFNAR. Mice were treated with either rmIL-22 i.p. (2 µg/20 mg bodyweight) or PBS on days 0, 2, 4, 6, and 8. A group of mice received anti-IFNAR i.p. (10 mg/kg bodyweight). All mice were terminated at day 10. **(B and C)** Histological evaluation of colonic section with representative pictures (B) and absolute quantification for H&E (C; *n* = 5/8/6/8/8). Bars, 500 µm (upper); 200 µm (lower). **(D–G)** Histological evaluation of small intestinal sections with representative pictures and absolute quantification for H&E (D and E) and TUNEL (F and G); *n* = 5/8/6/8/8). Bars, 100 µm. Results represent one experiment. Significance determined using two-tailed Student’s *t* test (C, E, and G) and expressed as the mean ± SEM. *, P < 0.05; ***, P < 0.001.

We next assessed the contribution of IFN-I signals to the IL-22–driven ileitis in *Atg16l1*^ΔIEC^ mice. In this experiment, mice received a slightly harsher DSS regimen of 2% DSS for a total of 5 d to also induce robust colonic inflammation. In addition, *Atg16l1*^fl/fl^ and *Atg16l1*^ΔIEC^ mice received rmIL-22 i.p. every other day for 10 d. To block the IFN-I pathway, one group of mice additionally received a blocking antibody against the common IFN α/β receptor (IFNAR; anti-IFNAR) at days 0, 2, 4, and 6 (Yang et al., 2015; Fig. 8 A). With the harsher regimen of DSS treatment, *Atg16l1*^ΔIEC^ mice displayed stronger histological signs of colonic inflammation compared with *Atg16l1*^fl/fl^ animals. While IL-22 treatment did not significantly affect the severity of colonic inflammation (Fig. 8, B and C), it significantly exacerbated the severity of ileal inflammation in *Atg16l1*^ΔIEC^ mice. Cotreatment of IL-22–stimulated *Atg16l1*^ΔIEC^ mice with anti-IFNAR antibody reduced the severity of small intestinal inflammation (Fig. 8, D and E), which was again associated with decreased cell death in the crypt region (Fig. 8, F and G). This indicates that IFN-I synergizes with IL-22 induced epithelial cell death and ileal inflammation in *Atg16l1*^ΔIEC^ mice. To further assess to which extent IFN-I, independently of IL-22, influences epithelial cell death in *Atg16l1*^ΔIEC^ in vitro, we stimulated *Atg16l1*^ΔIEC^ and *Atg16l1*^fl/fl^ intestinal organoids with IFN-β (1,000 U/ml) and assessed cell viability. IFN-β induced organoid cell death, which was significantly amplified in *Atg16l1*^ΔIEC^ organoids (Fig. S5, A and B). To confirm the hypothesis that IFN-I contributes to ileal inflammation in *Atg16l1*^ΔIEC^ mice independent of an exogenous IL-22 stimulus, we performed a 2% DSS colitis with either anti-IFNAR or with corresponding IgG control at days 0, 2, 4, and 6 (Fig. S5 C) in the without IL-22 injection. *Atg16l1*^ΔIEC^ mice displayed increased colonic inflammation, which was not significantly ameliorated by anti-IFNAR treatment (Fig. S5, D and E). However, and in line with our in vitro findings, treatment with anti-IFNAR antibody alone reduced small intestinal inflammation and epithelial cell death in *Atg16l1*^ΔIEC^ mice (Fig. S5, F–H).

### The IL-22–IFN-I axis is associated with clinical outcome upon anti-TNF therapy in IBD patients

**Figure 9.**
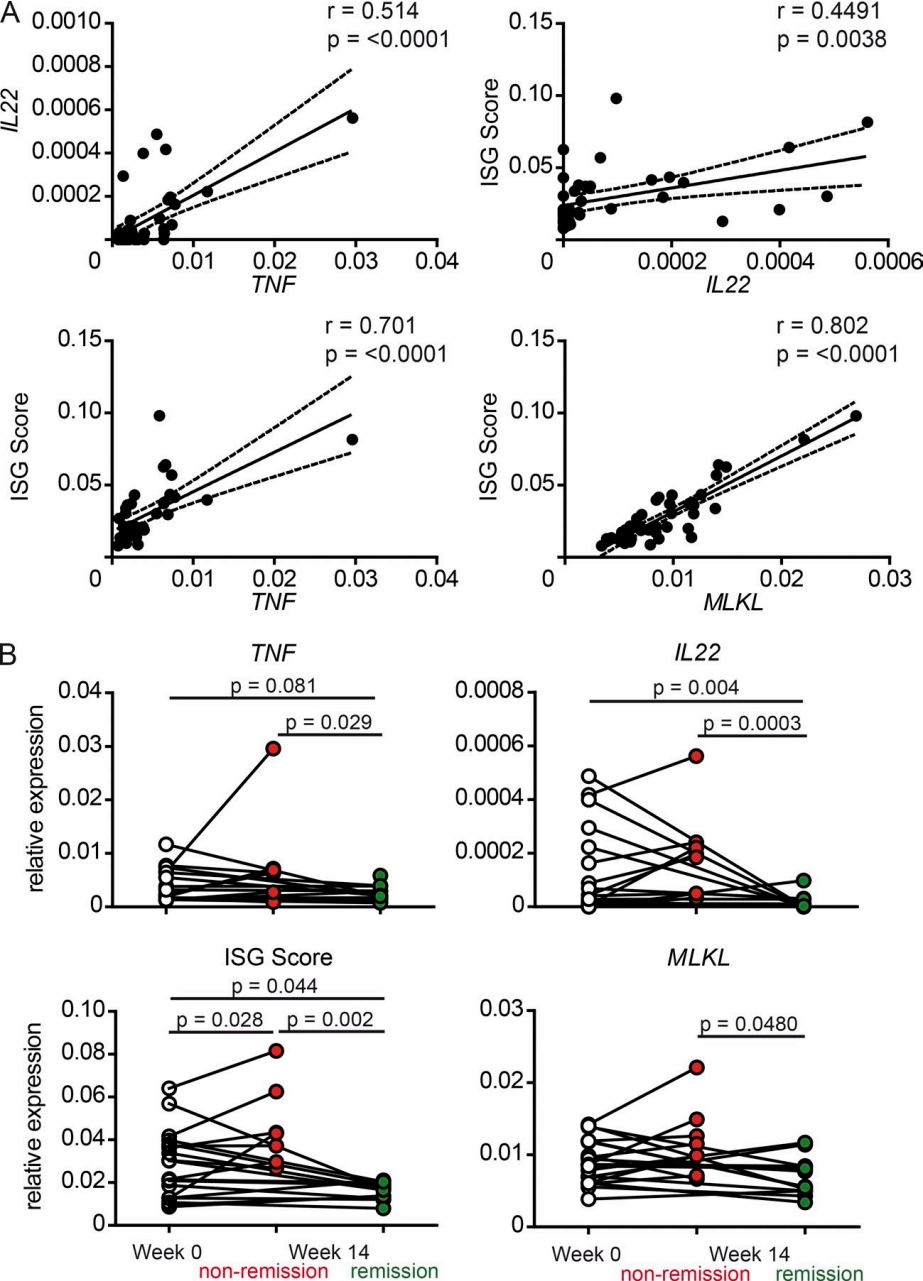
**The IL-22–IFN-I axis affects clinical outcome upon anti-TNF therapy in IBD patients. (A)** Linear regression of correlation of *IL22*, *TNF*, and *MLKL* or a composite score (Lübbers et al., 2013) of six IFN stimulatory genes (ISG) in sigmoid biopsies from IBD patients (*n* = 21). **(B)** Relative mRNA expression of *IL22, TNF, MLKL*, or a composite score of six ISG in sigmoid biopsies from human IBD patients before (week 0) or after anti-TNF therapy (week 14) clustered according to clinical remission status (remission: UC = 7, CD = 7; nonremission: CD = 1, UC = 6). Significance determined using Spearman test for correlation (A) or Mann-Whitney test (B) and expressed as the mean ± SEM.

Prompted by our murine in vitro and in vivo findings we elucidated whether the IL-22–ISG axis associates with disease severity in IBD. We collected sigmoid biopsies before (week 0) or after (week 14) anti-TNF antibody treatment induction from 21 IBD patients (*n* = 21; UC = 13 and CD = 8) and assessed mucosal mRNA expression by qPCR of *IL22*, *TNF*, and *MLKL* and a composite score of six IFN-I–inducible genes (*IFITM1*, *MXA*, *OAS3*, *IFIT1*, *IFI44L*, and *IFI16)*, termed “ISG score” hereafter, previously shown to robustly correlate with IFN-I signaling (Lübbers et al., 2013). Overall, *IL22* mRNA levels were positively correlated with *TNF* and *MLKL* expression, as well as the ISG score (Fig. 9 A). In contrast to baseline expression, only IBD patients achieving clinical remission after anti-TNF treatment (*n* = 21; remission: UC = 7 and CD = 7; nonremission: CD = 1 and UC = 6) displayed a significant reduction of mucosal *IL22*, *TNF*, and *MLKL* mRNA expression, as well as the ISG score levels (Fig. 9 B). Thus, our data indicate that the IL-22–ISG axis might be involved in therapy response to anti-TNF treatment in IBD patients.

## Discussion

The interplay of hypomorphic autophagy function and increased ER stress in intestinal epithelial cells is an important part of the etiology of human IBD. Our data now provide evidence for a surprising link of this interplay to intestinal epithelial IL-22 signaling. IL-22 normally plays an essential role in intestinal homeostasis (Sabat et al., 2014). However, under certain conditions, IL-22 signaling may evoke tissue damage (Wang et al., 2017), leads to halted epithelial expansion in vitro (Lindemans et al., 2015), and may instead aggravate inflammatory responses (Zheng et al., 2007). A molecular explanation of this dichotomous role has remained obscure. Our data indicate that unrestricted and excessive IL-22 signaling may indeed perpetuate intestinal inflammation, rather than resolve it. In our experiments, IL-22 effects on intestinal wound healing even in the autophagy-proficient setting are strongly context dependent. In epithelial scratch assays, we show that immediate IL-22 stimulation after scratching leads to significantly improved wound healing (cell migration and proliferation), whereas continuous IL-22 stimulation already before induction (pretreatment) impeded scratch closure. This is in line with reports showing time-restricted pulsatile IL-22 expression during intestinal wound healing (Huber et al., 2012). In active IBD, high numbers of IL-22 producing cells (Geremia et al., 2011) and elevated levels of *IL22* mRNA and IL-22 protein have been documented in several independent studies (Brand et al., 2006; Pelczar et al., 2016; Feagan et al., 2017; Nikolaus et al., 2017). It is difficult to infer exact mucosal concentrations due to the spatially restricted expression of IL-22; however, it seems likely that measurable elevation of serum concentrations in active IBD (in the range of up to 0.5 ng/ml; Nikolaus et al., 2017), as well as in vitro secretion capacity from biopsies from experimental models of intestinal inflammation (Bachmann et al., 2010), reflect local IL-22 levels, which may well reach the range of the concentrations (1–100 ng/ml) used in the present study. This is in line with another study (Lindemans et al., 2015) indicating that deleterious IL-22 effects may correlate with higher concentrations. Moreover, recombinant IL-22Fc has been used in concentrations of 120 µg/kg bodyweight in humans (Lekkerkerker et al., 2017), indicating that the in vivo concentrations used in our mouse studies are highly relevant if also therapeutic application of IL-22 is considered.

Deficiency of *Atg16l1* aggravates the detrimental effects of IL-22 signaling, characterized by excessive epithelial cell death in vitro and in vivo. We show that the concomitant induction of ISG and TNF is dependent on IL-22–induced activation of cGAS–STING signaling. While physiologically detectable after IL-22 stimulation of WT primary intestinal epithelial cells, the cGAS–STING response is strongly increased in the absence of functional *Atg16l1*. This indicates that *Atg16l1* may be either important for removal of active STING signaling complexes (Saitoh et al., 2009; Bartsch et al., 2017; Prabakaran et al., 2018) or may be involved in the generation of the endogenous activation principle, e.g., by aggravating the ER stress-mitochondria axis or impairing maintenance of DNA integrity, which may lead to the induction of nuclear DNA damage and dsDNA release (Liu et al., 2015; Qiang et al., 2016; Wang et al., 2017).

*Atg16l1*^ΔIEC^ mice exposed to the DSS even at low doses develop significant inflammation and epithelial cell death in the terminal ileum, although the irritant usually only elicits colonic inflammation. Of note, *Atg16l1*^ΔIEC^ mice display a significant degree of enhanced epithelial cell death in the small intestine even under baseline condition. Both phenotypes are aggravated upon cotreatment with IL-22. In our hands, the effect seemed to be restricted to the small intestine, as exogenous IL-22 was not able to potentiate DSS-induced inflammation in the colon. Corroborating the specific effect on small intestinal inflammation, IL-22 treatment also worsened spontaneous CD-like ileitis and epithelial cell death in *Atg16l1*^ΔIEC^*/Xbp1*^ΔIEC^ mice, which coincided with increased mRNA levels of *Tnf* and *Ifnb*. Our observations thus indicate a tissue specific interplay of ATG16L1-dependent autophagy, necroptosis, and IL-22 signaling in the small intestinal mucosa, which is in agreement of *Atg16l1*’s role as a CD risk gene with a predominant ileal phenotype (Durães et al., 2013). It must be noted that our study setup solely focuses on the interaction of IL-22 signaling with impaired autophagy and ER stress in the intestinal epithelial layer. It will be interesting to elucidate the interplay of ATG16L1 and IL-22 with other cell types, e.g., immune cells, that might critically modulate epithelial remodeling (Zheng et al., 2007; Aden et al., 2016).

Dysregulated necroptosis is an important driver of epithelial cell death in the gut and is an active principle in chronic inflammatory states including human IBD (Duprez et al., 2011; Günther et al., 2011; Welz et al., 2011). It has been recently reported that *Atg16l1*^ΔIEC^ intestinal organoids are more susceptible to TNF-induced necroptosis, which underscores the importance of a sufficient autophagy machinery for fine tuning of cell death pathways (Matsuzawa-Ishimoto et al., 2017). We here provide evidence that dysfunctional autophagy (specifically, genetic defects in *Atg16l1*) may transform regenerative IL-22 signaling into a stimulus for MLKL-dependent epithelial necroptosis. Our data indicate that IL-22–dependent necroptosis requires enhanced STING signaling as (1) IL-22–induced TNF and ISG induction is inhibited in *Sting*^gt^ intestinal organoids, and (2) IL-22–induced cell death in autophagy-deficient cells is rescued in *Sting*^gt^ and in *Mlkl*^−/−^ intestinal organoids.

STING activation in this scenario leads to a strong epithelial ISG response. Although it has been reported that specific IFN-I target genes can be directly activated without prior IFN-I secretion, canonical ISG induction is thought to require IFN-I signaling (Schneider et al., 2014). We provide evidence that such IFN-I signals might, either directly or indirectly, be involved in the observed cell death phenotype. (1) IFN-β leads to the induction of MLKL and TNF and cell death in epithelial organoids, which is aggravated in *Atg16l1-*deficient conditions. (2) Blocking the IFN-I receptor with a blocking anti-IFNAR antibody leads to amelioration of disease severity. However, it must be noted that epithelial cell death and ileal inflammation in *Atg16l1*^ΔIEC^ mice were also ameliorated in the DSS model without additional IL-22. This indicates that the pathological consequence of exacerbated IFN-I signaling in the autophagy-deficient situation is not strictly dependent on IL-22, although we cannot rule out endogenous IL-22 signaling in the DSS model. It must also be pointed out that most of the observations in the organoid experiments are based on mRNA levels, which in our hands are more reliable than assaying secreted protein levels in the supernatant, potentially due to Matrigel embedding and low cell density. Although we have clearly detected elevated IFN-β upon IL-22 stimulation in vivo in the spontaneous ileitis in *Atg16l1*^ΔIEC^*/Xbp1*^ΔIEC^ mice, the exact role of autocrine IFN-I hence remains to be elucidated. STING-dependent necroptosis might be potentiated through IFN-I from other cellular sources, but might also be influenced by an impact of ATG16L1 on IFN-I independent mechanisms of STING-induced cell death (Sze et al., 2013; Cerboni et al., 2017; Gaidt et al., 2017; Larkin et al., 2017).

Collectively, our data point to a crucial role of autophagy and ER stress in controlling IL-22 signaling at the ileal mucosal barrier. It is conceivable that a switch to excessive IFN-I and TNF induction and subsequent programmed cell death may indeed be protective in virus infections, where often autophagy and resolution of ER stress are impaired (Hernández et al., 2015; Kuriakose et al., 2016; Mouna et al., 2016). Under conditions of chronic intestinal inflammation and with clear genetic “hits” to the autophagic and ER stress machinery like in human IBD, such a fate of IL-22 signals may crucially contribute to a vicious circle of tissue damage and inflammation. With recombinant IL-22 fusion proteins currently being developed as potential therapy in human intestinal inflammation as GvHD (clinicaltrials.gov identifier: NCT02406651) and IBD (clinicaltrials.gov identifier: NCT02749630; Stefanich et al., 2018), this dichotomy may have important clinical implications.

## Materials and methods

### Antibodies and reagents

Recombinant murine and human IL-22, epidermal growth factor, and IL-10 were purchased from Peprotech; TM was purchased from Calbiochem; bafilomycin A1 was purchased from Enzo; rapamycin was purchased from Sigma Aldrich; and iSTAT3 inhibitor was purchased from EMD Millipore. Recombinant murine IFN-β was purchased from R&D. Antibodies targeting ATG16L1 (D6D5), γH2AX (Ser139; D17A3), STING (D2P2F), pTBK1 (Ser172; D52C2), TBK1 (D1B4), MLKL (D6W1K), pIRF-3 (Ser396; 4D4G), IRF-3 (D83B9; all from Cell Signaling Technology), pMLKL (Ser345; EPR9515(2); Abcam), β-ACTIN (Sigma Aldrich), and GAPDH (Santa Cruz) were used for immunoblot analysis. Neutralizing anti-TNF (MP6-XT22) antibody was purchased from Biolegend. For immunohistochemistry (IHC), antibodies against γH2AX (Ser139; Cell Signaling Technology) were applied. Anti-dsDNA (HYB331-01) antibody for immunofluorescence (IF) staining was purchased from Santa Cruz. Propidium iodide (PI) was purchased from BD Biosciences. In vivo IFN-I blockade was established using anti-IFNAR antibody (clone: MAR1-5A3; Biocxell), which was used as described before (Yang et al., 2015). Murine anti-TNF antibody was purchased from Biolegend.

### Cell culture

HT-29 and Caco-2 human colorectal adenocarcinoma cells were derived and authenticated from the Deutsche Sammlung von Mikroorganismen und Zellkulturen. Cell lines were seeded in 6-, 12-, or 24-well plates, respectively. Using the CRISPR/Cas9 technology, three independent *ATG16L1*-deficient Caco-2 cell clones were established. Cells were cultured in DMEM + 10% FCS or MEM + 20% FCS, until confluency. Before stimulation, cells were serum-starved by incubating DMEM + 1% FCS medium or MEM + 1% FCS for 24 h. Mycoplasma infection of cell lines were excluded by applying biweekly mycoplasma testing using Mycoalert (Lonza).

### Epithelial scratch assay

Scratch assays were performed as described before (Liang et al., 2007). In brief, using a P100 pipette tip, an epithelial wound was created by rapidly scratching a fully confluent cellular monolayer. A line was drawn on the bottom of each well using a scalpel to identify same wound areas directly after creating the wound and after 24 h of incubation. Epithelial wound closure was assessed by imaging using Zeiss Axio Vert.A1 and digital measurement of the cell free area using AxioVision LE (Zeiss) software. The relative wound closure is calculated as follows: *Relative wound closure* = 1 – (*wound area after 24 h* / *starting wound area*).

### Mice

Villin*(V)-cre*^+^; *Xbp1*^fl/fl^ (*Xbp1*^ΔIEC^) (Niederreiter et al., 2013), Villin*(V)-cre^+^*; *Atg16l1*^fl/fl^ (*Atg161*^ΔIEC^) (Adolph et al., 2013), Villin*(V)-cre*^+^; *Atg16l1*^fl/fl^*/Xbp1*^fl/fl^ (*Atg16l1*^ΔIEC^*/Xbp1*^ΔIEC^) (Adolph et al., 2013), Villin*(V)-cre*^+^; *Sting*^gt^, *Mda5*^−/−^, and *Mlkl*^−/−^, backcrossed for at least six generations on a *C57BL/6* background, were used at an age of 8–12 wk for all experiments with appropriate genotype littermate controls. *Cgas*^−/−^ were provided by A. Roers (Technical University Dresden, Dresden, Germany), *Il28r*^−/−^ were provided by P. Staeheli (University Hospital Freiburg, Freiburg, Germany), and *Irf3*^−/−^ were provided by M. Brinkmann (Helmholtz Centre for Infection Research, Braunschweig, Germany). For *Il28r*^−/−^ and *Irf3*^−/−^, *Sting*^gt^, *Mda5*^−/−^, and *Mlkl*^−/−^, nonlittermate *C57BL/6* animals at an age of 8–12 wk were used as controls animals and were declared as WT.

*Ormdl3*^fl/fl^ mice were generated in cooperation with Taconic. In brief, proximal loxP sites were inserted into *Ormdl3* gene on exon 2. A distal *loxP* site was introduced with an FRT flanked neomycin selection cassette within exon 4. The resultant mouse line was bred with deleter-mice constitutively expressing Flp recombinase to remove the neomycin selection cassette, creating an *Ormdl3*^f/+l^ mouse in which *Ormdl3* exons 2–4 were flanked by two *loxP* sites. After backcrossing onto *C57BL*/*6*, these mice were crossed with *V-cre^+^* mice resulting in *V-cre; Ormdl3*^fl/fl^ mice with intestinal-epithelial-cell-specific *Ormdl3* deletion (*Ormdl3*^ΔIEC^). *Ormdl3*^fl/fl^ mice were crossed with *Atg16l1*^ΔIEC^ mice to develop *V-cre^+^;Atg16l1*^fl/fl^*;Ormdl3*^fl/fl^ (*Atg16l1*^ΔIEC^/*Ormdl3*^Δ^*^IEC^*) mice.

All mice were maintained in a specific pathogen–free facility, and quarterly health report did not indicate presence of pathogenic bacteria, viruses, or protozoa. All *Atg16l1*^fl/fl^ colonies were norovirus free. Littermates were cohoused throughout the entire length of the experiments in a maximum genotype ratio of 1:2 (i.e., two fl/fl animals cohoused with a single ΔIEC). Mice were provided with food and water ad libitum and maintained in a 12-h light–dark cycle under standard conditions at Kiel University (*Atg161*^ΔIEC^, *Xbp1*^ΔIEC^, *Atg161*^ΔIEC^*/Ormdl3*^ΔIEC^, *Mlkl*^−/−^), the University of Cambridge (*Atg161*^ΔIEC^*/Xbp1*^ΔIEC^), University Hospital Bonn (*Sting*^gt^ and *Mda5*^−/−^), Technical University Dresden (*Cgas*^−/−^), University Hospital Freiburg (*Il28r*^−/−^), and Helmholtz Centre for Infection Research (*Irf3*^−/−^).

Tail or ear biopsy genomic DNA was used for genotyping of respective mouse strains. For experiments including application of recombinant murine IL-22 or DSS experiments equal numbers (minimum *n* = 5 per genotype; see figure legends for details) of male and female animals were used. Procedures involving animal care were conducted in conform to national and international laws and policies and appropriate permission. All experiments were performed in accordance with the guidelines for Animal Care of Kiel University and University of Cambridge (for experiments involving *Atg161*^ΔIEC^*/Xbp1*^ΔIEC^ mice).

### In vivo treatment of mice

*Atg16l1*^ΔIEC^ or *Atg16l1*^fl/fl^ mice were treated with either 2 µg/20 mg bodyweight recombinant murine IL-22 (Peprotech) or PBS i.p. for indicated time before being sacrificed for intestinal epithelial cell isolation as described elsewhere (Pickert et al., 2009). In addition, *Atg16l1*^ΔIEC^ or *Atg16l1*^fl/fl^ mice were supplied with 2% DSS (MP Biomedical) dissolved in autoclaved drinking water on day 1 for three consecutive days followed by 2 d of regular drinking water. Disease Activity Index was assessed as described previously (Siegmund et al., 2001). *Atg16l1*^ΔIEC^*/Xbp1*^ΔIEC^ or *Atg16l1*^fl/fl^*/Xbp1*^fl/fl^ mice were treated with either 2 µg/20 mg bodyweight recombinant murine IL-22 (Peprotech) or PBS i.p. every other day for 13 d (seven injections in total) before being sacrificed for intestinal epithelial cell isolation as described elsewhere (Pickert et al., 2009). For the anti-IFNAR antibody rescue experiment, *Atg16l1*^ΔIEC^ or *Atg16l1*^fl/fl^ mice were treated with anti-IFNAR antibody f (10 mg/kg bodyweight; clone: MAR1-5A3) at days 0, 2, 4, and 6 of DSS colitis. Mice were additionally supplied with 2% DSS dissolved in autoclaved drinking water from day 0–5 followed by 5 d of regular drinking water.

### Histopathological analyses of murine small intestinal tissue

Post mortem, the entire small intestine was excised and separated longitudinally into two equal parts. The longitudinal section was rolled up, starting with the distal part thereby having the distal ileum at the very inner layer and the proximal intestine at the very outer layer. The entire specimen was fixed in 10% formalin. Paraffin sections were cut and stained with H&E. Histological scoring was performed in a blinded fashion by two independent observers. The histological score displays the combined score of inflammatory cell infiltration and tissue damage as described elsewhere (Adolph et al., 2013).

### IHC and IF

For IHC and IF staining, 5-µm sections of paraffin-embedded colon/ileum Swiss rolls were deparaffinized with Tyrol substitute (Roth).

For IHC, slides were incubated in citrate buffer for 3 min and subsequently blocked with blocking serum (Vectastain) for 20 min. Primary antibodies were incubated overnight. Sections were washed, incubated with secondary antibodies and DAB substrate (Vectastain ABC kit). For TUNEL assay, slides were subjected to Apop Tag Plus Peroxidase In situ Apoptosis Detection kit (Merck Millipore) according to manufacturer’s protocol.

For IF staining of tissue, paraffin-embedded sections were blocked in PBS containing 5% BSA and 0.2% Triton X-100 for 30 min after removal of citric buffer and before incubation of a primary antibody overnight at 4°C. Secondary antibodies conjugated with fluorophores were added after washing steps with PBS for 45 min. Then, tissue was counterstained with DAPI and DRAQ5 and then mounted with fluorescence mounting medium (DAKO).

For IF staining of Caco-2 cells, cells were fixed on cover slides using 4% paraformaldehyde for 30 min at room temperature. After washing steps, cells were permeabilized for 3 min at room temperature using PBS containing 1% Triton X-100 and 5% BSA. Cells were blocked using 5% goat serum for at least 60 min at room temperature. The further staining procedure was identical to the IF protocol for tissues.

For quantification of dsDNA or pTBK-1 a minimum of 100 crypts/intestine were assessed in each treatment group and statistical evaluation showing the mean number of dsDNA^+^ or pTBK^+^ cells per treatment group. Slides were visualized by an AxioImager Z1 microscope (Zeiss; Germany). Pictures were captured by a digital camera system (AxioCam HrC/HrM, Zeiss). Measurements were made using a semi-automated image analysis software (AxioVision version 08/2013).

### Cultivation of murine intestinal organoids

Mouse intestinal organoids were cultivated as described before (Sato et al., 2009). In brief, small intestine was removed and cleared of intestinal content by flushing the intestine with HBSS. After removal of residual fat and Peyer’s patches, the intestine was cut longitudinally and then laterally in pieces of 0.5 cm. Intestinal pieces were incubated in ice-cold PBS + 10 nM EDTA for 10 min for four times, intermitted by vigorous shaking and replacement with fresh PBS + 10 nM EDTA after every shaking process. The crypt suspension was then strained through a 100-µm strainer, followed by a spin with 1,200 rpm at 4°C. Pure epithelial crypts were suspended in Matrigel (BD Bioscience) to a concentration of 5–10 crypts/1 µl Matrigel, embedded in 24-well plates and cultivated in intestinal stem cell medium (IntestiCult Organoid Growth Medium [Mouse], StemCell Technologies, Inc.) based on previously described organoid medium containing murine EGF, murine Noggin, and human R-Spondin 1 (Sato et al., 2009). Medium was changed every other day, and organoids were stimulated after 3–6 d of cultivation after passage.

### Epithelial cell death assay

Organoids derived from *Atg16l1*^ΔIEC^ or *Atg16l1*^fl/fl^ mice were seeded into 96-well plates. For FACS-based cell death assay using PI, organoids were dissociated into single cells using TrypLE Express (Thermo Fisher Scientific). Cells were then incubated in PI for 10 min before FACS assay using FACScalibur (BD Bioscience). 10,000 cells were gated excluding doublets or nondissociated cell groups. The fraction of cells positive for PI staining was considered as dead cells (Dannappel et al., 2014). For microscopic assessment, 1 µl PI was added to 500 µl medium overnight. Medium was removed and matrigel droplets containing the organoids were washed with PBS. Merged images were then captured using multidimensional imaging with brightfield and RFP fluorescence filter using Zeiss Axio Vert.A1.

### Immunoblot analysis

Cells were lysed using SDS-based DLB buffer + 1% Halt Protease inhibitor cocktail (Thermo Fisher Scientific) before heating on 95°C for 5 min and followed by sonification for 5 s twice. To remove cell remnants, lysates were centrifuged at 16,000 *g* for 15 min at 4°C. For protein extraction of organoids, Matrigel was removed by several centrifugation steps at 4°C followed by lysis as described above. Afterward, equal amounts of lysates containing Laemmli buffer were heated at 95°C and electrophoresed on 12% polyacrylamide gels under standard SDS-PAGE conditions before being transferred onto a polyvinylidene fluoride membranes (GE Healthcare). Protein loaded membranes were blocked with 5% milk in TBS-T, incubated with primary antibody overnight and with HRP-conjugated secondary antibody for 1 h at indicated concentrations. Proteins were detected using Amersham ECL Prime Western Blot Detection Reagent (GE Healthcare).

### ELISA

Supernatants from stimulated cell cultures were analyzed for indicated protein concentration using ELISA kit according to manufacturer’s protocol for IL-8 (Thermo Fisher Scientific) and murine TNF and murine Cxcl10 (Peprotech).

### cDNA synthesis and gene expression analysis

mRNA isolation of PBS washed cells, snap­-frozen tissue, and PBS­-washed Matrigel containing organoids was performed using RNEasy kit (Qiagen). cDNA synthesis was performed using RevertAid Premium cDNA Synthesis kit (Fermentas) according to manufacturer’s protocol. Gene expression was subjected to the cDNA samples using SYBR Green qPCR or TaqMan assays, which were purchased from Applied Biosystems. Reactions were performed on the Applied Biosystems 7900HT Fast Real-Time PCR System (Applied Biosystems), and relative transcript levels were determined using *Actb* (TaqMan and SYBR Green) and *Gapdh* (SYBR Green) as a housekeeper. Primer sequences were retrieved designed using Primer3 software version 0.4.0 (Untergasser et al., 2012), except for those individually referenced (for sequences see Table S7). TaqMan Probes were derived from Applied Biosystems (for TaqMan probe IDs see Table S8).

### Transcriptomics analysis

RNaseq was conducted on small intestinal organoids derived from *Atg16l1*^Δ^*^IEC^* and *Atg16l1^fl/fl^* mice (*n* = 4) that were treated with recombinant murine IL-22 (10 ng/ml) or controls. Samples were sequences on Illumina HiSeq3000 using Illumina total RNA stranded TruSeq protocol. An average of ∼28 million 150-nt paired-end reads was sequenced for each sample. Raw reads were preprocessed using cutadapt (Martin, 2011) to remove adapter and low quality sequences. RNaseq reads were aligned to the mm10/Ensemble (GrCm38) reference genome with TopHat2 (Trapnell et al., 2012). Data were deposited in the NCBI Gene Expression Omnibus under GSE119354. Gene expression values of the transcripts were computed by HTSeq (Anders et al., 2015). Differential gene expression levels were analyzed and visualized by the Bioconductor package DESeq2 (Love et al., 2014). The overall effect of IL-22 treatment of organoid cells on WT and *Atg16l1*^Δ^*^IEC^* mice was obtained by multifactorial experimental design of DESeq2. Likelihood ratio test was used to assess the significant differentially expressed genes (DEG) of WT/KO-IL-22 treatment interaction of *Atg16l1*^Δ^*^IEC^* mice (P value <0.01). Venn diagrams were drawn using VennDiagram package in R (Chen and Boutros, 2011). To gain insight into the nature of DEGs uniquely expressed in *Atg16l1*^fl/fl^ and *Atg16l1*^Δ^*^IEC^* mice upon IL-22 treatment (up- and down-regulated), GO terms obtained within the category of biological processes using the InnateDB database (Breuer et al., 2013).

### Candidate validation in human IBD samples

For validation of ISG, *IL22*, *TNF*, and *MLKL* signatures under anti-TNF therapy we recruited a total of 22 IBD patients including 13 patients with UC and 9 patients with CD. The study was approved by the ethics committee of Kiel University (A 124/14) and subjects provided written informed consent. We included patients with active colonic disease who received vedolizumab or infliximab for induction of remission in standard medical care. Indication and choice of treatment were not part of the study protocol. Patients were investigated 24 h before initiation of treatment and at weeks 2, 6, and 14 after initiation of treatment. At each time point, 60 ml of peripheral blood were obtained and a sigmoidoscopy was performed, in which up to eight biopsies were taken from the sigmoid colon. Peripheral blood and biopsies from the sigmoid colon of patients were obtained within 24 h before initiation of treatment and immediately before administration of infliximab at weeks 2, 6, and 14. CD patients with a Harvey-Bradshaw Index (HBI) of ≤4 were considered to be in remission, those with ≥5 to have active disease. Patients with UC and a total Mayo Score of ≤2 (bleeding 0 and endoscopy ≤1) were considered to be in remission, those with ≥3 to have active disease. To create an ISG score we assessed the expression of a total of six known ISG (*IFITM1*, *MX1*, *OAS3*, *IFIT1*, *IFI44l*, and *IFI16*), which are shown to correlate strongest with type I IFN expression in human, as described previously (Lübbers et al., 2013). An ISG score was calculated by averaging the relative expression of these genes.

### Generation of CRISPR guided deletion of ATG16L1^−/−^ Caco-2 cells

A CRISPR/Cas9 vector (Thermo Fisher Scientific) was created according to the manufacturer’s instructions, using a dsDNA oligo targeting ATG16L1 (5′-GCAGCAAGTGACATGTCGT-3′). The vector was transfected with Lipofectamine 3000 into Caco-2 cells, and clonal cell lines were generated. Knockout clones were selected by verifying absence of ATG16L1 protein by Western blot analysis.

### Statistical information

Statistical analysis was performed using GraphPad Prism (version 4.0) for Windows software (GraphPad Software). No data are excluded for analyses. Statistical significance was evaluated by Mann–Whitney *U-*test for nonparametric data or the Student's *t* test for parametric data unless indicated otherwise. P values <0.05 were considered statistically significant.

### Online supplemental material

Fig. S1 shows that IL-22 increased proinflammatory signals in the context of increased ER stress or impaired autophagy. Fig. S2 shows that IL-22–induced ER stress, controlled by STAT3 and autophagy, impairs intestinal regeneration. Fig. S3 shows additional data on cell death assay and transcriptome analysis of IL-22–treated *Atg16l1*^ΔIEC^ organoids. Fig. S4 shows that the interplay of ER stress and autophagy is linked to increased IL-22–induced STING/IFN-I activation. Fig. S5 shows that the impact of IFN-I signaling on ileitis in *Atg16l1*^ΔIEC^ mice is independent of exogenous IL-22 administration. Table S1 shows up-regulated genes in untreated *Atg16l1*^fl/fl^ intestinal organoids compared to A*tg16l1*^ΔIEC^. Table S2 shows down-regulated genes in untreated *Atg16l1*^fl/fl^ intestinal organoids compared to A*tg16l1*^ΔIEC^. Table S3 shows genes uniquely up-regulated in *Atg16l1*^fl/fl^ + IL-22–treated intestinal organoids. Table S4 shows genes uniquely down-regulated in *Atg16l1*^fl/fl^ + IL-22–treated intestinal organoids. Table S5 shows genes uniquely up-regulated in A*tg16l1*^ΔIEC^ + IL-22–treated intestinal organoids. Table S6 shows genes uniquely down-regulated in A*tg16l1*^ΔIEC^ + IL-22–treated intestinal organoids. Table S7 shows SYBR Green primers used for quantitative real-time PCR (qRT-PCR). Table S8 shows TaqMan probes used for quantitative real-time PCR.

## Supplementary Material

Supplemental Materials (PDF)

Table S1

Table S2

Table S3

Table S4

Table S5

Table S6
