# Supplementary material for: ATG16L1 orchestrates interleukin-22 signaling in the intestinal epithelium via cGAS–STING
Source: J Exp Med. 2018 Nov 5;215(11):2868–86. doi: 10.1084/jem.20171029 (PMC6219748; doi:10.1084/jem.20171029)
Supplement: Supplemental Materials (PDF) [file JEM_20171029_sm.pdf]

Supplemental material

Aden et al., https://doi.org/10.1084/jem.20171029

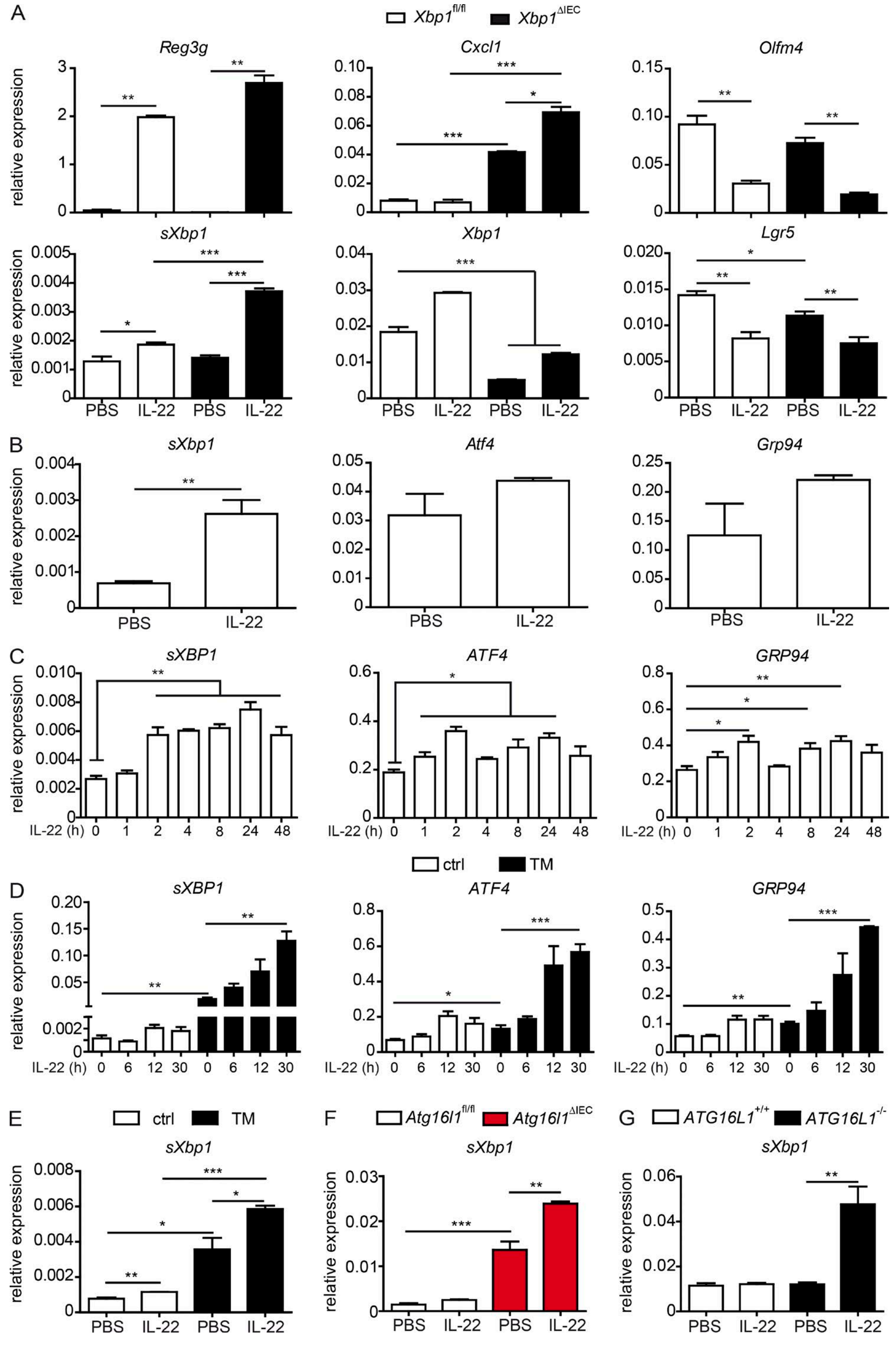

Figure S1. **IL-22 increases cell death and proinflammatory signals in the context of increased ER stress or impaired autophagy (related to Fig. 1).** **(A)** Gene expression of *Reg3g*, *Cxcl1*, *sXbp1*, *Xbp1*, *Olfm4*, and *Lgr5* in small intestinal organoids ($Xbp1^{fl/fl}$, $Xbp1^{\Delta IEC}$) treated with rmIL-22 (100 ng/ml) for 24 h (n = 3 each) was quantified by qPCR. **(B)** *C57BL/6J* mice were i.p. injected with rmIL-22 (2 µg) for 24 h, small intestinal epithelial crypts were isolated, and gene expression of ER stress markers *sXbp1*, *Atf4*, and *Grp94* was quantified by qPCR (n = 3 each). **(C)** HT-29 cells were stimulated with rhIL-22 (100 ng/ml) for indicated time points, and the expression of the ER stress markers *sXBP1*, *ATF4*, and *GRP94* was assessed by qPCR (n = 3 each). **(D)** HT-29 cells were prestimulated with rhIL-22 (100 ng/ml) for indicated time points; medium was changed and stimulated with TM (1 µg/ml) or DMSO for another 6 h. Expression of *sXBP1*, *ATF4*, and *GRP94* was assessed by qPCR (n = 4 each). **(E)** Intestinal organoids were pretreated with rmIL-22 (10 ng/ml) for 24 h, succeeded by stimulation with TM (0.5 µg/ml) for 6 h. Expression of *sXbp1*, *Atf4* and *Grp94* was assessed by qPCR (n = 3 each). **(F)** $Atg16l1^{fl/fl}$ and $Atg16l1^{\Delta IEC}$ intestinal organoids were stimulated with rmIL-22 (100 ng/ml) for 24 h. Expression of *sXbp1* was assessed by qPCR (n = 3 each). **(G)** $ATG16L1^{+/+}$ and $ATG16L1^{-/-}$ Caco-2 cells were stimulated with rhIL-22 (100 ng/ml) for 24 h. Expression of *sXBP1* was assessed by qPCR (n = 3 each). Results represent two (A,B, and E–G) or four (C and D) independent experiments. Significance determined using two-tailed Student's t test and expressed as the mean ± SEM. *, $P < 0.05$; **, $P < 0.01$; ***, $P < 0.001$.

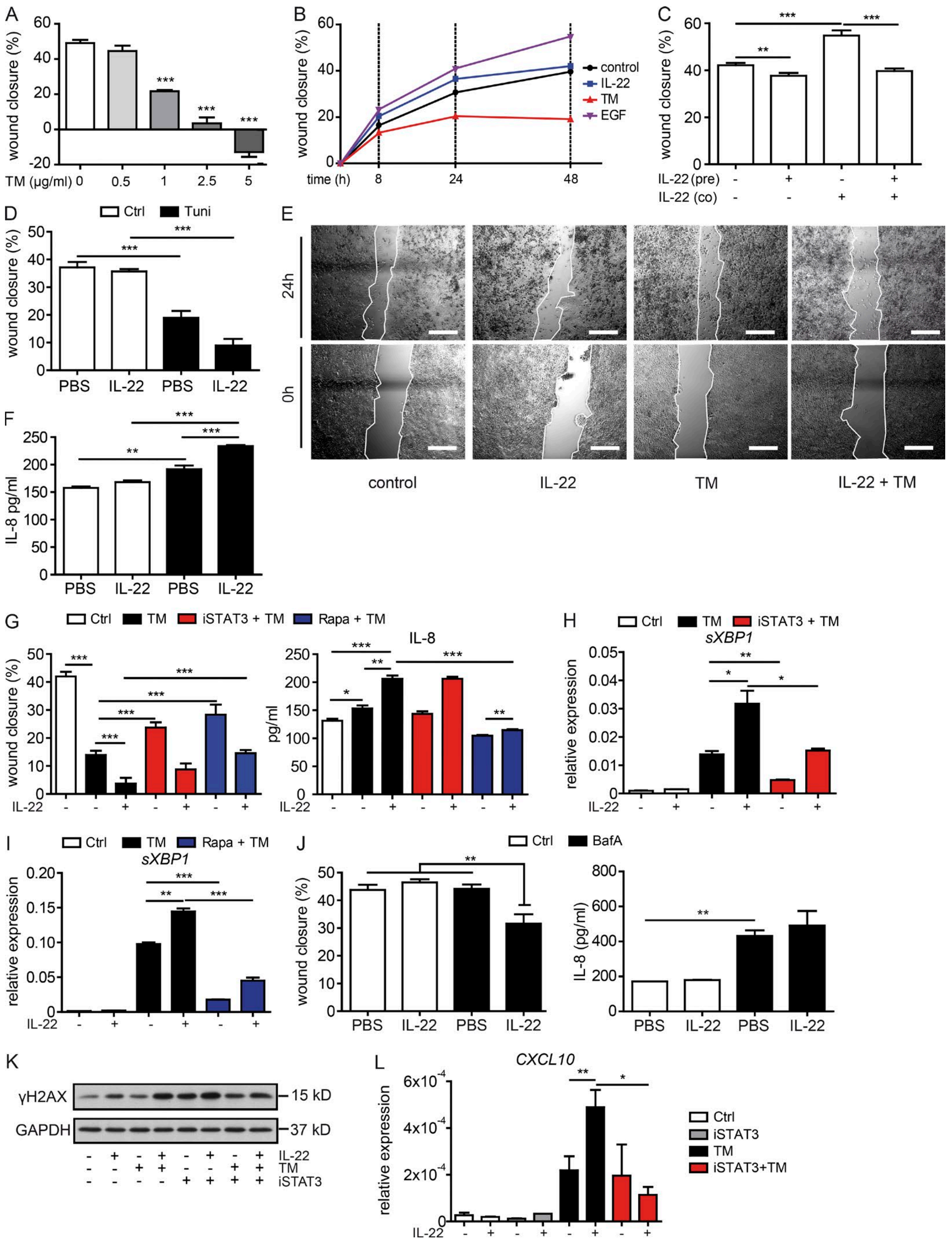

Figure S2. **IL-22 induced ER stress impairs intestinal regeneration (related to Fig. 1). (A)** HT-29 monolayers were wounded with coinciding addition of increasing doses of TM for 24 h. Relative wound closure was analyzed after 24 h (*n* = 6 each). **(B)** HT-29 cellular monolayers were established, wounded, and treated with rhIL-22 (100 ng/ml), TM (1 µg/ml), or rhEGF (100 ng/ml). Relative wound closure was assessed after 8, 24, and 48 h (*n* = 4 each). **(C)** HT-29 monolayers were either stimulated with rhIL-22 (100 ng/ml) 24 h prior (pre) or coinciding (co) or both to wound induction. Relative wound closure was assessed 24 h after wounding (*n* = 4 each). **(D–F)** HT-29 monolayers were prestimulated with rhIL-22 for 24 h, medium changed, and wounded. After wounding, cells were treated with or without TM (1 µg/ml), and wound healing was assessed after 24 h (*n* = 4 each). Statistical analyses of relative wound closure (D), corresponding representative pictures are shown (*n* = 4 each; bars, 100 µm; E), and IL-8 ELISA of supernatants (F). **(G)** HT-29 cells were stimulated with rhIL-22 (100 ng/ml) in the presence or absence of STAT3 inhibitor S3I-201 (iSTAT3, 50 µM) or rapamycin (Rapa, 10 nM) for 24 h, medium was changed, and cells were stimulated with TM for another 24 h (*n* = 4 each). Statistical analyses of relative wound closure and IL-8 ELISA of supernatants were shown. **(H and I)** HT-29 cells pretreated for 24 h with IL-22 and iSTAT3 (H) or Rapamycin (I), followed by medium change and stimulation with TM (1 µg/ml) for 6 h (*n* = 4 each), and expression of *sXBP1* was assessed by qPCR. **(J)** HT-29 cells were pretreated with IL-22 for 24 h, followed by medium change and stimulation with bafilomycin A (BafA, 5 nM) for 24 h (*n* = 4 each). Relative wound healing and corresponding IL-8 secretion are shown. **(K and L)** HT-29 cells were stimulated with rhIL-22 (100 ng/ml) in the presence or absence of STAT3 inhibitor S3I-201 (iSTAT3, 50 µM) and TM (1 µg/ml) for 24 h. Western blot against antibodies detecting γH2AX and GAPDH is shown (K). Isolated mRNA was subjected to qPCR detecting *CXCL10* expression (L). Results represent three (A–J) or two (K and L) independent experiments. Significance determined using two-tailed Student's *t* test and expressed as the mean ± SEM. *, P < 0.05; **, P < 0.01; ***, P < 0.001.

https://doi.org/10.1084/jem.20171029

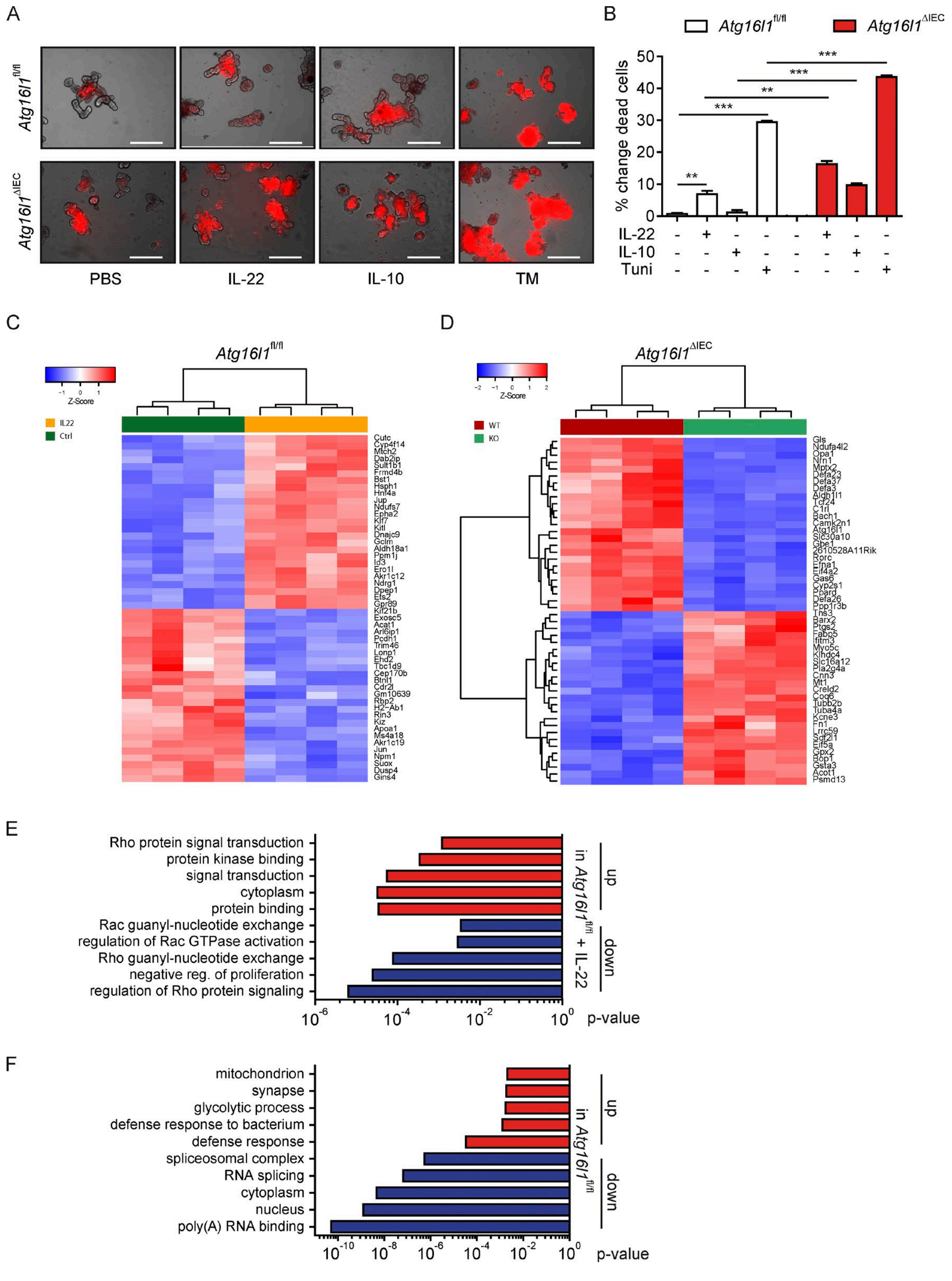

Figure S3. **Additional data on cell death assay and organoid sequencing (related to Figs. 1 and 2). (A)** Representative pictures of intestinal organoids derived from *Atg16l1*$^{fl/fl}$ and *Atg16l1*$^{\Delta IEC}$ mice and treated with rmIL-10 (100 ng/ml) or TM (500 ng/ml) for 24 h stained with propidium iodide (PI). Bars, 200 µm. **(B)** Flow cytometry assessment of dead cells from intestinal organoids (*Atg16l1*$^{fl/fl}$, *Atg16l1*$^{\Delta IEC}$) stimulated with rmIL-10 (100 ng/ml) or TM (500 ng/ml) for 24 h using PI (n = 4 each). **(C)** Heat map showing top 25 uniquely up- and 25 down-regulated genes in *Atg16l1*$^{fl/fl}$ organoids treated with rmIL-22 (10 ng/ml). **(D)** Heat map showing top 25 up- and 25 down-regulated genes in untreated *Atg16l1*$^{fl/fl}$, *Atg16l1*$^{\Delta IEC}$ intestinal organoids. **(E)** GO-term analysis using the input of top 250 uniquely up- and 250 down-regulated genes in *Atg16l1*$^{fl/fl}$ intestinal organoids stimulated with rmIL-22 (10 ng/ml) for 24 h. **(F)** GO-term analysis using the input of top 250 up- and 250 down-regulated genes in untreated *Atg16l1*$^{fl/fl}$ compared with untreated *Atg16l1*$^{\Delta IEC}$ intestinal organoids. Results represent two (A and B) independent experiments. Significance determined using two-tailed Student's t test and expressed as the mean ± SEM. **, P < 0.01; ***, P < 0.001.

https://doi.org/10.1084/jem.20171029

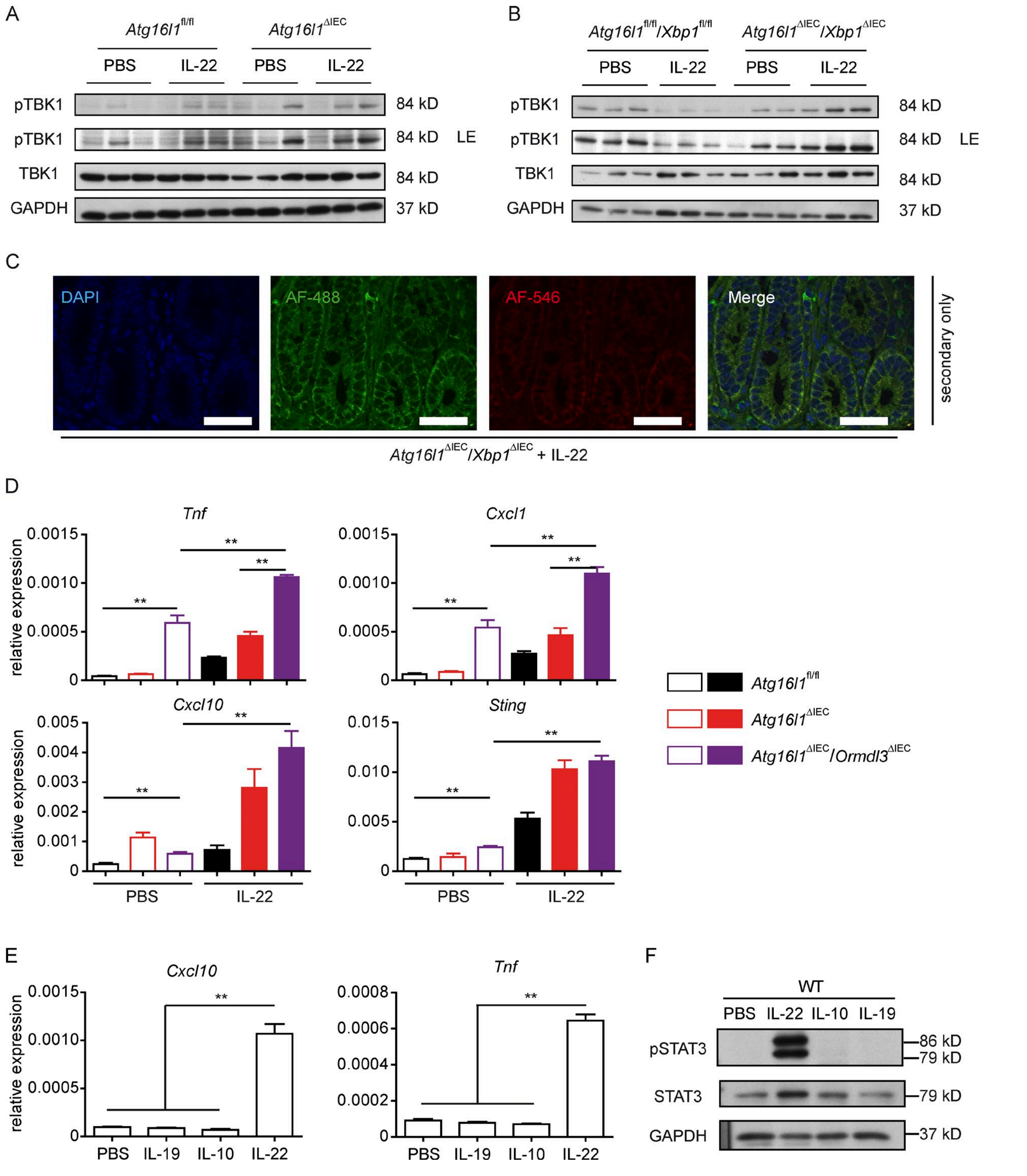

Figure S4. **Interplay of ER stress and autophagy is linked to increased IL-22–induced STING/IFN-I activation (related to Figs. 4 and 5). (A and B)** Isolated intestinal epithelial cells from IL-22–treated *Atg16l1*$^{fl/fl}$ *or Atg16l1*$^{\Delta IEC}$ mice were subjected to immunoblot analysis and probed against pTBK1, TBK1, and GAPDH (A; *n* = 3/3/3/3). Isolated intestinal epithelial cells from IL-22–treated *Atg16l1*$^{fl/fl}$/*Xbp1*$^{fl/fl}$ and *Atg16l1*$^{\Delta IEC}$/*Xbp1*$^{\Delta IEC}$ mice were subjected to immunoblot analysis and probed against pTBK1, TBK1, and GAPDH (B; *n* = 3/3/3/3). LE: Longer exposure. **(C)** Negative control for determination of pTBK1 and E-cadherin specificity. Small intestinal sections from *Atg16l1*$^{\Delta IEC}$/*Xbp1*$^{\Delta IEC}$ + IL-22–treated mice were anti-rabbit Alexa Fluor 546 (red, secondary to pTBK1) and anti-mouse Alexa Fluor 488 (green, secondary to E-cadherin) and counterstained with DAPI. Bars, 100 µm. **(D)** Gene expression of *Cxcl1*, *Cxcl10*, *Tnf*, and *Sting* in small intestinal organoids (*Atg16l1*$^{fl/fl}$, *Atg16l1*$^{\Delta IEC}$, *Atg161*$^{\Delta IEC}$/*Ormdl3*$^{\Delta IEC}$) treated with rmIL-22 (100 ng/ml) for 24 h (*n* = 4). **(E)** Gene expression of *Cxcl10, Tnf* in small intestinal organoids (*C57BL/6*) treated with rmIL-22, rmIL-10, and rmIL-19 (all 100 ng/ml) for 24 h (*n* = 4 each). **(F)** Western blot of C57BL/6 WT intestinal organoids stimulated IL-22, IL-10, and IL-19 (100 ng/ml) for 30 min. Protein lysates were probed against pSTAT3, STAT3, and GAPDH as loading control. Results are representative for *n* = 2 biological replicates (C and D). Significance determined using two-tailed Student's *t* test (C and D) and expressed as the mean ± SEM. **, P < 0.01.

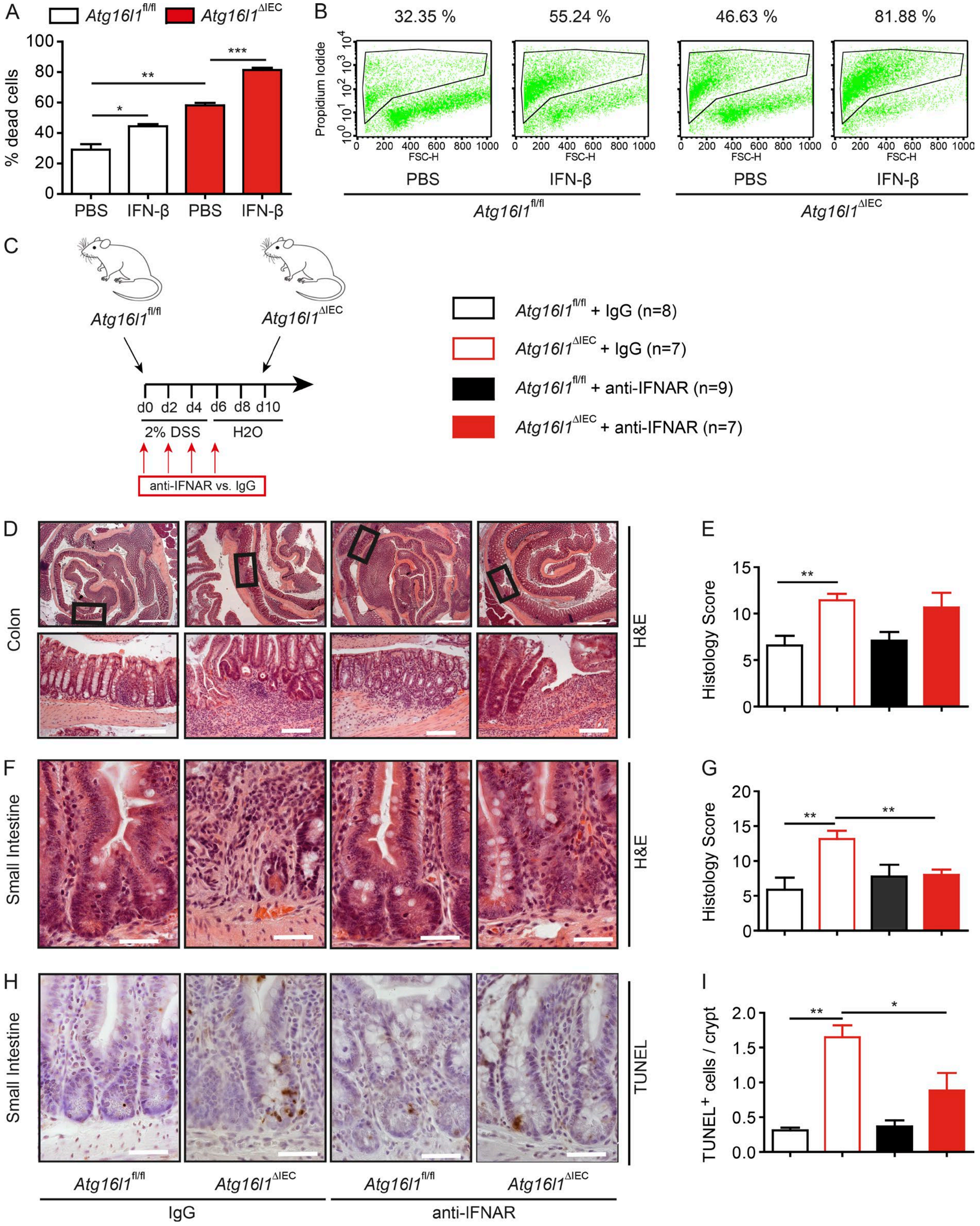

Figure S5. **Impact of IFN-I signaling on ileitis in *Atg16l1*$^{\Delta IEC}$ mice independent of exogenous IL-22 administration (related to Fig. 8). (A)** Flow cytometry assessment of dead cells from intestinal organoids (*Atg16l1*$^{fl/fl}$, *Atg16l1*$^{\Delta IEC}$) stimulated with rmIFN-β (1,000 U/ml) for 24 h using PI (*n* = 3 per group). **(B)** Representative FACS plots of PI-stained dissociated epithelial cells from intestinal organoids derived from *Atg16l1*$^{fl/fl}$ and *Atg16l1*$^{\Delta IEC}$ mice and treated with rmIFN-β (1,000 U/ml) for 24 h. Data are representative for a minimum of *n* = 2 individual experiments (A and B). **(C)** Stimulation scheme of *Atg16l1*$^{fl/fl}$ and *Atg16l1*$^{\Delta IEC}$ mice treated with anti-IFNAR antibody or corresponding IgG control (*n* = 8/7/9/7). Mice received i.p. anti-IFNAR antibody or IgG (10 mg/kg bodyweight) at day 0, 2, 4, and 6. All mice were sacrificed at day 10. **(D and E)** Histological evaluation of colonic section with representative pictures (D) and absolute quantification for H&E (E). Bars: 500 µm (upper); 200 µm (lower). **(F–I)** Histological evaluation of small intestinal sections with representative pictures and absolute quantification for H&E (F and G) and TUNEL (H and I). Bars, 100 µm. Significance determined using two-tailed Student's *t* test (A, E, G, and I) and expressed as the mean ± SEM. *, P < 0.05; **, P < 0.01; ***, P < 0.001.

Tables S1–S6 are included as separate text files. Tables S7 and S8 are included as separate Word files. Table S1 shows upregulated genes in untreated Atg16l1$^{fl/fl}$ intestinal organoids compared to Atg16l1$^{\Delta IEC}$. Table S2 shows downregulated genes in untreated Atg16l1$^{fl/fl}$ intestinal organoids compared to Atg16l1$^{\Delta IEC}$. Table S3 shows genes uniquely upregulated in Atg16l1$^{fl/fl}$ + IL-22–treated intestinal organoids. Table S4 shows genes uniquely downregulated in Atg16l1$^{fl/fl}$ + IL-22–treated intestinal organoids. Table S5 shows genes uniquely upregulated in Atg16l1$^{\Delta IEC}$ + IL-22–treated intestinal organoids. Table S6 shows genes uniquely downregulated in Atg16l1$^{\Delta IEC}$ + IL-22–treated intestinal organoids. Table S7 shows SYBR Green primers used for qRT-PCR. Table S8 shows TaqMan probes used for qRT-PCR.

References

Ather, J.L., K.A. Fortner, R.C. Budd, V. Anathy, and M.E. Poynter. 2013. Serum amyloid A inhibits dendritic cell apoptosis to induce glucocorticoid resistance in CD4(+) T cells. *Cell Death Dis.* 4:e786. https://doi.org/10.1038/cddis.2013.327

Chen, X., D. Iliopoulos, Q. Zhang, Q. Tang, M.B. Greenblatt, M. Hatziapostolou, E. Lim, W.L. Tam, M. Ni, Y. Chen, et al. 2014. XBP1 promotes triple-negative breast cancer by controlling the HIF1α pathway. *Nature.* 508:103–107. https://doi.org/10.1038/nature13119

Déry, M.A., and A.C. LeBlanc. 2017. Luman contributes to brefeldin A-induced prion protein gene expression by interacting with the ERSE26 element. *Sci. Rep.* 7:42285. https://doi.org/10.1038/srep42285

Fan, S., B. Liu, L. Sun, X.B. Lv, Z. Lin, W. Chen, W. Chen, Q. Tang, Y. Wang, Y. Su, et al. 2015. Mitochondrial fission determines cisplatin sensitivity in tongue squamous cell carcinoma through the BRCA1-miR-593-5p-MFF axis. *Oncotarget.* 6:14885–14904. https://doi.org/10.18632/oncotarget.3659

Fribley, A., K. Zhang, and R.J. Kaufman. 2009. Regulation of apoptosis by the unfolded protein response. *Methods Mol. Biol.* 559:191–204. https://doi.org/10.1007/978-1-60327-017-5_14

Hasan, M., C.S. Fermaintt, N. Gao, T. Sakai, T. Miyazaki, S. Jiang, Q.Z. Li, J.P. Atkinson, H.C. Morse III, M.A. Lehrman, and N. Yan. 2015. Cytosolic Nuclease TREX1 Regulates Oligosaccharyltransferase Activity Independent of Nuclease Activity to Suppress Immune Activation. *Immunity.* 43:463–474. https://doi.org/10.1016/j.immuni.2015.07.022

Iwakoshi, N.N., A.H. Lee, P. Vallabhajosyula, K.L. Otipoby, K. Rajewsky, and L.H. Glimcher. 2003. Plasma cell differentiation and the unfolded protein response intersect at the transcription factor XBP-1. *Nat. Immunol.* 4:321–329. https://doi.org/10.1038/ni907

Kaser, A., A.-H. Lee, A. Franke, J.N. Glickman, S. Zeissig, H. Tilg, E.E.S. Nieuwenhuis, D.E. Higgins, S. Schreiber, L.H. Glimcher, and R.S. Blumberg. 2008. XBP1 links ER stress to intestinal inflammation and confers genetic risk for human inflammatory bowel disease. *Cell.* 134:743–756. https://doi.org/10.1016/j.cell.2008.07.021

Lian, L.H., Q. Jin, S.Z. Song, Y.L. Wu, T. Bai, S. Jiang, Q. Li, N. Yang, and J.X. Nan. 2013. Ginsenoside Rh2 Downregulates LPS-Induced NF- κ B Activation through Inhibition of TAK1 Phosphorylation in RAW 264.7 Murine Macrophage. *Evid. Based Complement. Alternat. Med.* 2013:646728. https://doi.org/10.1155/2013/646728

Luo, M., H. Wang, Z. Wang, H. Cai, Z. Lu, Y. Li, M. Du, G. Huang, C. Wang, X. Chen, et al. 2017. A STING-activating nanovaccine for cancer immunotherapy. *Nat. Nanotechnol.* 12:648–654. https://doi.org/10.1038/nnano.2017.52

Ma, Y., Y. Shimizu, M.J. Mann, Y. Jin, and L.M. Hendershot. 2010. Plasma cell differentiation initiates a limited ER stress response by specifically suppressing the PERK-dependent branch of the unfolded protein response. *Cell Stress Chaperones.* 15:281–293. https://doi.org/10.1007/s12192-009-0142-9

Niedźwiecka, K., M. Dyląg, D. Augustyniak, G. Majkowska-Skrobek, M. Cal-Bąkowska, Y.H. Ko, P.L. Pedersen, A. Goffeau, and S. Ułaszewski. 2016. Glutathione may have implications in the design of 3-bromopyruvate treatment protocols for both fungal and algal infections as well as multiple myeloma. *Oncotarget.* 7:65614–65626. https://doi.org/10.18632/oncotarget.11592

Pokatayev, V., N. Hasin, H. Chon, S.M. Cerritelli, K. Sakhuja, J.M. Ward, H.D. Morris, N. Yan, and R.J. Crouch. 2016. RNase H2 catalytic core Aicardi-Goutières syndrome-related mutant invokes cGAS-STING innate immune-sensing pathway in mice. *J. Exp. Med.* 213:329–336. https://doi.org/10.1084/jem.20151464

Slepak, T.I., M. Tang, V.Z. Slepak, and K. Lai. 2007. Involvement of endoplasmic reticulum stress in a novel Classic Galactosemia model. *Mol. Genet. Metab.* 92:78–87. https://doi.org/10.1016/j.ymgme.2007.06.005

Wang, Q., H. Mora-Jensen, M.A. Weniger, P. Perez-Galan, C. Wolford, T. Hai, D. Ron, W. Chen, W. Trenkle, A. Wiestner, and Y. Ye. 2009. ERAD inhibitors integrate ER stress with an epigenetic mechanism to activate BH3-only protein NOXA in cancer cells. *Proc. Natl. Acad. Sci. USA.* 106:2200–2205. https://doi.org/10.1073/pnas.0807611106
